# A BMP7 Variant Inhibits Tumor Angiogenesis *In Vitro* and *In Vivo* through Direct Modulation of Endothelial Cell Biology

**DOI:** 10.1371/journal.pone.0125697

**Published:** 2015-04-28

**Authors:** Courtney M. Tate, Jacquelyn Mc Entire, Roberto Pallini, Eliza Vakana, Lisa Wyss, Wayne Blosser, Lucia Ricci-Vitiani, Quintino Giorgio D’Alessandris, Liliana Morgante, Stefano Giannetti, Luigi Maria Larocca, Matilde Todaro, Antonina Benfante, Maria Luisa Colorito, Giorgio Stassi, Ruggero De Maria, Scott Rowlinson, Louis Stancato

**Affiliations:** 1 Discovery Research, Eli Lilly and Company, Indianapolis, United States of America; 2 Department of Neurosurgery, Università Cattolica del Sacro Cuore, Rome, Italy; 3 Department of Hematology, Oncology and Molecular Medicine, Istituto Superiore di Sanità, Rome, Italy; 4 Institute of Human Anatomy, Università Cattolica del Sacro Cuore, Rome, Italy; 5 Institute of Pathology, Università Cattolica del Sacro Cuore, Rome, Italy; 6 Surgical and Oncological Sciences, University of Palermo, Palermo, Italy; 7 Regina Elena National Cancer Institute, Rome, Italy; University of Bari Medical School, ITALY

## Abstract

Bone morphogenetic proteins (BMPs), members of the TGF-β superfamily, have numerous biological activities including control of growth, differentiation, and vascular development. Using an *in vitro* co-culture endothelial cord formation assay, we investigated the role of a BMP7 variant (BMP7v) in VEGF, bFGF, and tumor-driven angiogenesis. BMP7v treatment led to disruption of neo-endothelial cord formation and regression of existing VEGF and bFGF cords *in vitro*. Using a series of tumor cell models capable of driving angiogenesis *in vitro*, BMP7v treatment completely blocked cord formation. Pre-treatment of endothelial cells with BMP7v significantly reduced their cord forming ability, indicating a direct effect on endothelial cell function. BMP7v activated the canonical SMAD signaling pathway in endothelial cells but targeted gene knockdown using shRNA directed against SMAD4 suggests this pathway is not required to mediate the anti-angiogenic effect. In contrast to SMAD activation, BMP7v selectively decreased ERK and AKT activation, significantly decreased endothelial cell migration and down-regulated expression of critical RTKs involved in VEGF and FGF angiogenic signaling, VEGFR2 and FGFR1 respectively. Importantly, in an *in vivo* angiogenic plug assay that serves as a measurement of angiogenesis, BMP7v significantly decreased hemoglobin content indicating inhibition of neoangiogenesis. In addition, BMP7v significantly decreased angiogenesis in glioblastoma stem-like cell (GSLC) Matrigel plugs and significantly impaired *in vivo* growth of a GSLC xenograft with a concomitant reduction in microvessel density. These data support BMP7v as a potent anti-angiogenic molecule that is effective in the context of tumor angiogenesis.

## Introduction

Bone morphogenetic proteins (BMPs) belong to the larger TGF-β family of extracellular ligands and are involved in a multitude of developmental and homeostatic processes [1]. BMPs bind to BMP type I and type II STK membrane receptors to transduce signals through SMADs 1/5/8 and SMAD 4, along with non-SMAD signaling pathways [2,3]. Recently the BMP family members have been implicated in tumor progression, most notably acting as suppressors of tumor-initiating precursor cells (TICs), also referred to as cancer stem cells (CSCs) [4–6]. In a wide range of tumor histologies, CSCs may give rise to the proliferating tumor mass as well as metastatic cells [7–9], with the importance of these cells in tumorigenesis further expanded to include their pivotal role in angiogenesis and vascular mimicry [10]. There have been a number of additional reports linking BMP family members and their cognate receptors with a role in angiogenesis [11]. BMP9 and BMP10 are high affinity ligands for the type I receptor ALK1, specifically expressed on endothelial cells and possibly playing a role in endothelial cell dynamics [12]. BMP4 induced human umbilical vein endothelial cell sprouting, migration and tube formation [13,14] along with paracrine effects on tumor vasculature in malignant melanoma [15]. These observations as well our previous results showing that a BMP7 variant (BMP7v) was not only able to reduce glioblastoma brain invasion but also associated angiogenesis [16] led us to further investigate the mechanism of this angiogenic inhibition.

We used an *in vitro* endothelial cord formation assay, a surrogate of angiogenesis, to investigate the role of BMP7v signaling in VEGF, bFGF, and tumor-driven angiogenesis [17,18]. To our knowledge BMP7v is the only systemically available member of this family reported to be efficacious in preclinical cancer models, having been engineered to address poor PK issues that plague the wild type protein [16], and therefore represents a significant step forward in its potential use in treating cancer. Unlike wild type BMP7, BMP7v was able to avoid inhibition by circulating endogenous inhibitors such as noggin, chordin and chordin-like 2 via reduced binding. BMP7v treatment led to disruption of neo-angiogenic cord formation *in vitro* (and a reduction in hemoglobin content *in vivo*) and regression of existing VEGF and bFGF established cords *in vitro*. Using a series of tumor cell models capable of secreting soluble pro-angiogenic factors and therefore driving angiogenesis *in vitro*, BMP7v treatment completely blocked cord formation. In addition, pre-treatment of endothelial cells with BMP7v led to a significant reduction in their cord forming ability, indicating a direct effect on endothelial cell function. BMP7v activated the canonical SMAD signaling pathway in endothelial cells but, surprisingly, SMAD signaling was *not* required to mediate the anti-angiogenic effect. BMP7v decreased endothelial cell migration and down regulated expression of the RTKs involved in VEGF and FGF angiogenic signaling, VEGFR2 and FGFR1 respectively, while selectively reducing ERK and AKT activation. As previously reported [16], BMP7v was able to significantly delay *in vivo* GSLC xenograft growth with a concomitant and significant reduction in CD31 positive endothelial cells and microvessel density (MVD). Consequently, BMP7v directly modulates key signaling components within angiogenic pathways to elicit an anti-angiogenic phenotype and thereby likely contributing to its anti-tumor effects [16].

## Materials and Methods

### Cell culture and BMPs

Tumor cell lines (U-87-MG, PC-3, 786–0, MDA-MB-231, SK-OV-3) were obtained from ATCC (Manassas, VA) in May 2004, July 2005, April 2010, February 2005, September 2003 respectively and grown according to ATCC guidelines, A-2780 were obtained from NCI in March 2005, LXFA-629 NSCL adenocarcinoma cells were obtained from Oncotest (Freiburg, Germany) in July 2011, and were maintained in RPMI 1640 medium (Invitrogen, Grand Haven, NY) supplemented with 10% heat-inactivated FBS (Invitrogen) and 1% glutamine (Invitrogen). Tumor cells above were stored within a central cell bank that performs cell line characterizations. GSLCs were isolated from primary tumors between December 2003 and November 2005 and grown as neurospheres as previously described (19). GSCLs were characterized by flow cytometry and immunohistochemistry as previously described (19). Characterization of the banked cell lines was done last between December 2012 and August 2013 by a third-party vendor (RADIL, PCR profiling) for contamination of bacteria and viruses along with verification to be of human origin without mammalian interspecies contamination. The alleles for 9 different genetic markers were used to determine that the banked cells from ATCC and NCI matched the genetic profile that has been previously reported. Endothelial cord forming cells (ECFCs; Lonza, Walkersville, MD, January 2008) and adipose derived stem cells (ADSCs, Zen-Bio, Research Triangle Park, NC, March 2010) were maintained in EGM-2 MV media (Lonza) containing an additional 5% embryonic stem cell FBS (Invitrogen) for ECFCs. ADSCs and ECFCs were tested for their cord forming ability and pathogen tested as described above but no further method of authentication was done. ECFCs were grown in collagen-coated flasks; all other cells used tissue culture treated flasks in a humidified atmosphere at 37°C and 5% CO_2_. All cells were passaged for fewer than 4 months (ECFCs less than 7 passages, ADSCs less than 4 passages) before new cultures were initiated from frozen cells. BMP4, BMP9, and BMP10 were from R&D Systems (Minneapolis, MN); WT BMP7 and a modified version, BMP7v were manufactured as previously described [16].

### *In vitro* Cord Formation Assay

ADSCs (Zen-Bio) were plated at 75,000 cells/well into 96-well HTS Transwell receiver plates (tumor-driven; Corning, Tewksbury, MA) or 50,000 cells/well (growth factor-driven) into 96-well black poly-D lysine coated plates (BD Biosciences, San Jose, CA), and tumor cells were plated at 25,000 cells/well in 96-well HTS Transwell plates (Corning) in co-culture MCDB-131 media (Invitrogen) supplemented with L-ascorbic acid 2-phosphate, dexamethasone, tobramycin, insulin (Sigma-Aldrich, St. Louis, MO), and cell prime r-transferrin AF (Millipore, Billerica, MA) for 24 hours. ADSC media was removed and 6,000 (tumor-driven) or 5,000 (growth factor-driven) ECFCs per well were over-seeded. Treatment with PBS or 2 nM BMP7v occurred 4 hours following ECFC plating, for 96 hours. Where applicable, 10 ng/ml VEGF or bFGF (Invitrogen) were added simultaneously with PBS or BMP7v. Cells were directly fixed for 10 min with 3.7% formaldehyde (Sigma Aldrich) followed by ice-cold 70% ethanol for 30 min at 25°C. Cells were rinsed once with PBS, blocked for 30 min with 1% BSA, and immunostained for 1 hour with antiserum directed against CD31 (R&D Systems, Cat# AF806, sheep polyclonal) diluted to 1 μg/ml in 1% BSA. Cells were washed 3 times with PBS and incubated for 1 hour with 5 μg/ml donkey α-sheep-Alexa-488 (Molecular Probes, Grand Island, NY), α-Smooth Muscle Actin Cy3 conjugate (1:200, Sigma-Aldrich, Cat# C6198, mouse monoclonal), and 200 ng/ml Hoechst 33342 (Molecular Probes) in 1% BSA, washed with PBS, then imaged using the cord formation algorithm on the Cellomics ArrayScan VTI at a magnification of 5X for neo-cord formation or 4X for established cords (Thermo Fisher Scientific, Pittsburgh, PA). For the established cord assay, ADSCs/ECFCs were treated with 10 ng/ml growth factor (VEGF or bFGF) for 96 hours prior to addition of PBS or 100 ng/ml BMP7v.

### Endothelial tube formation assay

Human umbilical vein endothelial cells (HUVECs) (Lonza) were grown in EGM-2 MV media. HUVECs, pretreated with BMP4 (2 nM) and BMP7v (100 ng/ml) were plated (70,000 cells/well) in Matrigel-coated 24 well plates (BD Biosciences), and incubated for 5 hours at 37°C. Tube formation was evaluated by phase-contrast microscopy, and cord length was measured using Image J analysis software (NIH).

### Immunofluorescence

Cells were immunostained for CD31 (R&D Systems, Cat# AF806, sheep polyclonal), Ki67 (Millipore, Cat# AB9260, rabbit polyclonal), VEGFR2 (Cell Signaling Technology, Danvers, MA, Cat# 2479, rabbit monoclonal), FGFR1 (Cell Signaling Technology, Cat# 9740, rabbit monoclonal), or the In Situ Cell Death Detection Kit (Roche, Indianapolis, IN) per manufacturer’s recommendations, and 5 μg/ml goat α-rabbit-Alexa-647 (Invitrogen), or 5 μg/ml donkey α-sheep-Alexa-488 (Invitrogen) and 200 ng/ml Hoechst 33342, then imaged using the Target Activation algorithm on the VTI at an image magnification of 20X.

### Quantitative PCR analysis

HUVECs or ECFCs (8000 cells/well) were plated in collagen I coated 96-well plates. Medium was removed 16 hours later, and BMP or vehicle was added in media with 100 μg/ml BSA. RNA was harvested 24 hours later via Qiagen RNeasy kit including the DNAse treatment step per manufacturer’s instructions. RNA (500 ng) was converted to cDNA using Life Technologies cDNA archive kit per manufacturer’s instructions. Quantitative PCR analysis was performed with the following Applied Biosystems (Grand Island, NY) Taqman primer/probes: c-kit Hs00174029_m1, FGFR1 Hs00915142_m1, VEGFR2 Hs00911710, Kit ligand Hs00295067_s1, MCP-1 Hs00234140_m1, MMP-1 Hs00899658_m1, PECAM-1 Hs00169777_m1, PlGF Hs01119262_m1, β-actin 4333762T, GAPDH 4326317E, PGK1 5333765T, and PPIA 4333763T.

### ELISA analysis

Cells were cultured as above for qPCR analysis for 72 hours. Cell culture supernatants were collected and debris was removed by centrifugation. Supernatants were applied undiluted to ELISAs (R&D Systems) per manufacturer’s instructions.

### ECFC migration

Assays were performed as previously described [19]. Briefly, cells were plated in media containing 10% FBS on collagen I coated Oris Cell Migration plates containing seeding stoppers. Stoppers were removed 16 hours later, and treatments were added in Angiokit Optimised Growth Medium (Cell Systems Biology, Ontario). Medium was removed 24 hours later, and cells were fixed with Prefer (Anatech, Battle Creek, MI) for 30 minutes at RT, permeabilized with PBS containing 0.1% Triton X-100 for 15 minutes, washed twice with PBS and incubated with 50 μl of 15 μM propidium iodide (Sigma-Aldrich). Images were captured on the Acumen Explorer with a defined migration area.

### Western blot analysis

SDS (1%) whole-cell protein extracts were created, briefly sonicated, and protein was quantified using the Bradford method. Protein (25 μg) was subjected to electrophoresis on 4–20% pre-cast Tris-glycine gradient gels (Invitrogen), transferred to nitrocellulose (Invitrogen), blocked with 5% blotting grade blocker (Biorad, Hercules, CA) in Tris-buffered saline containing 0.1% tween (TBST), probed with primary antiserum, washed with TBST, and incubated with HRP-labelled secondary antibody. Membranes were washed with TBST, and signal was detected by ECL (Thermo Fisher Scientific). Antiserum directed against VEGFR2 (Cat# 2479, rabbit monoclonal), FGFR1 (Cat# 9740, rabbit monoclonal), pERK1/2 (Thr202/Tyr204) (Cat# 4370, rabbit monoclonal), total ERK1/2 (Cat# 4695S, rabbit monocolonal), pSAPK/JNK (Thr183/Tyr185) (Cat# 9251, rabbit polyclonal), pSMAD1 (Ser463/465)/pSMAD5 (Ser463/465)/pSMAD8 (Ser426/428) (Cat# 9511, rabbit polyclonal), pCRAF (Ser338) (Cat#9427, rabbit monoclonal), pMEK1/2 (Ser217/221) (Cat# 9154, rabbit monoclonal), total SMAD4 (Cat# 9515, rabbit polyclonal) (all from Cell Signaling Technology), phospho-p38 MAPK (Thr180/Tyr182) (Epitomics, Burlingame, CA, Cat# 1229–1, rabbit monoclonal), and β-actin (Santa-Cruz Biotechnology, Santa Cruz, CA, Cat# sc-47778, mouse monoclonal) were diluted (1:1000) with 5% blotting grade blocker in TBST. Densitometry was performed using Image J analysis software.

### shRNA-mediated knockdown of SMAD4 in ECFCs

ECFCs were transduced (MOI 9) with individual clone (non-target control, SCH202V) or equivalent amounts of five pooled clones of MISSION shRNA lentiviral SMAD4 transduction particles (TRCN0000010321, TRCN0000010322, TRCN0000010323, TRCN0000040028, TRCN0000040030) in growth media containing 8 μg/ml protamine sulfate for 72 hours prior to selection with 2.5 μg/ml puromycin (all from Sigma-Aldrich).

### *In vivo* Matrigel Plug Angiogenesis Assay

ADSCs (2 x 10^6^) and ECFCs (0.5 x 10^6^) were mixed on ice with 200 μl growth factor reduced Matrigel (BD Biosciences) and injected into the flanks of athymic nude female mice (Harlan, Indianapolis, IN). Cells in Matrigel were mixed with 100 ng BMP7v or 100 ng BMP4, or mice were dosed twice daily by intraperitoneal injection of BMP4, BMP7, or twice daily by oral gavage with sunitinib (25 mg/kg) 4 hours prior to cell implantation. Five days post implant, implants were removed, flash frozen, and hemoglobin was quantified using the QuantiChrom Hemoglobin Assay Kit (Bioassay, Hayward, CA) as previously described [17].

### GSLC Xenografts

Four-week-old male nude mice (HDS-athymic nude mice, Charles Rives, Milan, Italy) were used for the *in vivo* model of angiogenesis. The experiments with animals were approved by the Ethical Committee of the Catholic University School of Medicine, Rome. Green fluorescent protein (GFP) expressing GSLC1 and GSLC28 cells were harvested, washed twice, and resuspended in cold PBS at the concentration of 2 x 10^3^ cells per ml. Cells in 100 μl of PBS were mixed with 100 μl of Matrigel (BD Biosciences) on ice, and the mixture was implanted by subcutaneous injection. Treatment with BMP7v (1 μg per day) [16] was initiated one week after grafting. BMP7v (1 μg in 100 μl of saline) was injected intraperitoneally once daily for 3 weeks (5 days per week; total 15 injections). Control mice were intraperitoneally injected with an equal volume of saline.

Implants were removed 4 weeks post-grafting, stored in 30% sucrose buffer overnight at 4°C, and cryotome sectioned at 20 μm. IHC was performed as previously described [16]. Immunodetection was performed using the avidin biotin complex peroxidase method (LSAB Dako, Golstrup) using freshly made diaminobenzidine as chromogen. Alternate sections were stained with H&E for morphological analysis. Immunofluorescence was performed at 4°C on free floating sections rinsed with PBS. All primary antibody solutions were prepared in PBS containing 0.3% Triton X-100, and incubated with the sections overnight. Sections were incubated using antisera directed against CD31 (1:50, Santa Cruz Biotechnology, Santa Cruz, CA, Cat# sc-1506, goat polyclonal) or α-Smooth Muscle Actin (SMA; 1:200, Novus Biologicals, Cambridge, UK, Cat# NB600-531, rabbit polyclonal). After 3 PBS washes, sections were incubated for 1 hour at RT with Cy-3 conjugated anti-rabbit secondary antibody (1:400 Jackson Immuno Research Laboratories, West Grove, PA). Sections were mounted on slides with Vectashield mounting medium (Vector, Burlingame, CA). Images were obtained with a Zeiss LSM 510 META confocal laser scanning microscope (Zeiss, Milan). For quantitative analyses of angiogenesis, 10 separate fields were randomly selected/tissue section both in the periphery and in the center of the implants. “Periphery” = area lying between the subcutaneous fat and the outlying GFP-expressing GSLCs. “Center” = the area containing GFP-expressing GSLCs. Representative images were acquired using confocal microscopy and evaluated using the Zeiss AxioVision 4.4 dedicated software (Zeiss, Oberkochen, Germany).

### GSLC tumor efficacy study

Athymic nude mide (4–6 weeks; Charles River) were implanted subcutaneously with 5 x 10^5^ GSLC1 cells as described above [10,20]. Mice were kept under pathogen-free conditions in positive-pressure cabinets (Tecniplast Gazzada, Varese). Tumor diameter was measured using callipers and calculated as the mean value between the shortest and the longest diameters. Treatment with BMP7v (1 and 0.1 μg) or an equal volume of saline (controls) was initiated when the xenografts reached 9–13 mm in mean diameter and was administered by intraperitoneal injection every other day for 3 weekly cycles. Mice were sacrificed three weeks post-last dose, tumors excised; formalin fixed, paraffin-embedded sections (4 μm thick) were mounted on positive-charged glass slides. For antigen retrieval, deparaffinised and rehydrated sections were treated with citric acid buffer (pH 6.0) or with EDTA (pH 8.0), 2 x 5 minutes at 750 W, followed by inhibition of endogenous peroxidase with 3% H_2_O_2_ for 5 minutes. Sections were incubated with primary antibody and visualized using the avidin–biotin–peroxidase complex per manufacturer’s instructions. 3,3’-Diaminobenzidine was used as the enzyme substrate to observe specific antibody localization and Mayer hematoxylin was the nuclear counterstain. Samples were stained at least twice with high reproducibility. The histological pattern of tumor xenografts was assessed on H&E stained sections. The proliferative potential was assessed by Ki67 immunostaining (Dako, M7240, clone MIB-1, mouse monoclonal). Mitotic index (MI) was determined as the percentage of positive cell nuclei relative to the total number of cells in 20 high power fields. The expression of nestin, GFAP, and βIII tubulin, was assessed with anti-nestin (Santa Cruz Biotechnology, Cat# sc-23927, clone 10c2, mouse monoclonal), anti-GFAP (Millipore, Cat# AB5804, rabbit polyclonal), and anti-βIII tubulin (Millipore, Cat# CBL412X, clone TU-20, mouse monoclonal) antibodies. The expression of the endothelial phenotype was determined by CD31 (1:50, Santa Cruz Biotechnology, Santa Cruz, CA, Cat# sc-1506, goat polyclonal) immunostaining.

For CD31 and MVD quantitation, 5 μm thick paraffin-embedded sections were de-waxed, hydrated and heated with antigen retrieval. Sections were then treated with 3% H_2_O_2_ for 10 min, incubated with 10% human serum for 20 min and subsequently exposed to antibodies against mouse/human CD31 (Novocastra, Milan, NCL-CD31-1A10, mouse monoclonal) and human CD31 (Dako, clone JC70A, mouse monoclonal) or isotype-matched controls at appropriate dilutions using Antibody Diluent (Dako, S3022), at 4°C overnight. After washing, biotinylated secondary antibody against mouse immunoglobulin was applied for 30 min at RT (Dako, LSAB2 System-HRP, K0675). The slides were rinsed and incubated with streptavidin-biotin-peroxidase for 30 min. All sections were counterstained using AEC substrate and counter-stained with hematoxylin. MVD was determined by the number of microvessels positive for CD31 from images randomly selected from the tumor stroma. Positive vessels were counted from 3 fields and the average count/field was reported.

### Statistical analysis

Statistical significance was assessed by a two-tailed Student *t* test with equal variance compared to data obtained for PBS controls (*in vitro*) or control groups (*in vivo*). Statistical significance was assigned to *p* values <0.05.

## Results

### BMP7v reduced endothelial cord formation

**Fig 1:**
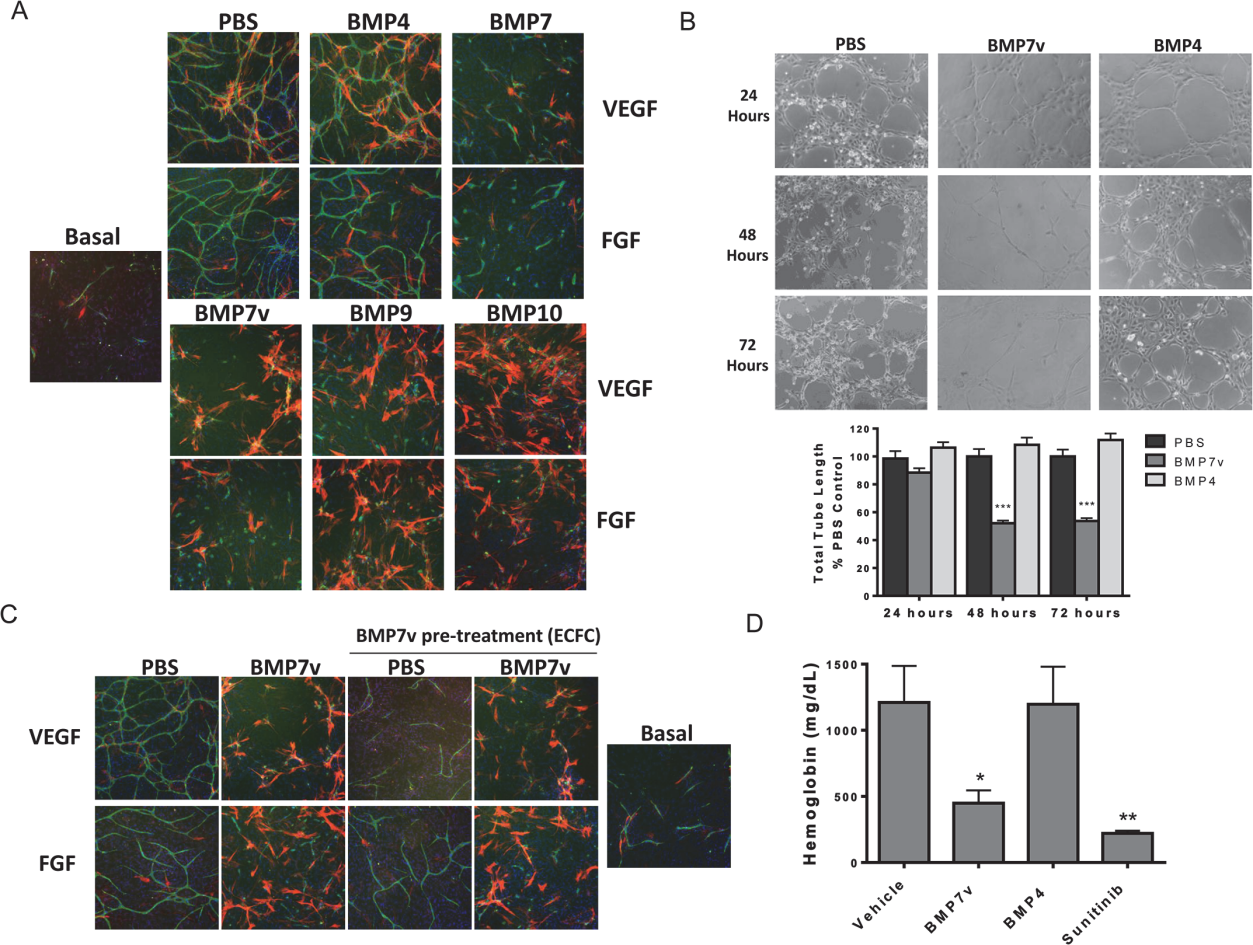
BMP7v reduced VEGF, bFGF, and Matrigel-driven cord formation. (A) The ADSC/ECFC co-culture was unstimulated (basal) or stimulated with 10 ng/ml VEGF or bFGF and treated simultaneously with PBS or 2 nM BMP4, BMP7, BMP7v, BMP9, or BMP10 for 96 hours prior to immunohistochemistry for CD31 (green), α-smooth muscle actin (red), and Hoechst 33342 to stain all nuclei (blue). Representative images (5X magnification) are shown, BMP7, BMP7v, BMP9, and BMP10 demonstrated statistically significant (***, *p<*0.001) differences in connected tube area compared to PBS controls. (B) HUVECs were pre-treated for 24, 48, or 72 hours with PBS, 2 nM BMP4, or 2 nM BMP7v then plated onto Matrigel for 5 hours. Representative images (10X magnification) are shown, graphs represent mean ± SEM from three independent experiments, and asterisks denote statistically significant (***, *p<*0.001) differences compared to PBS controls. (C) ECFCs were pre-treated with PBS or 100 ng/ml BMP7v for 24 hours then plated into the cord formation assay. The ADSC/ECFC co-culture was then unstimulated (basal) or stimulated with 10 ng/ml VEGF or bFGF for 96 hours prior to treatment with PBS or 100 ng/ml BMP7v for 72 hours and immunohistochemistry for CD31 (green), α-smooth muscle actin (red), and Hoechst 33342 to stain all nuclei (blue). Representative images (5X magnification) are shown, all samples treated with BMP7v displayed statistically significant (***, *p<*0.001) differences compared to PBS controls. (D) ADSC/ECFC cell mixture in Matrigel was treated with PBS, BMP7v (100 ng or 30 ng), or BMP4 (100 ng) and co-implanted subcutaneously into the flanks of athymic nude mice (8 mice per treatment group). Oral dosing of a subset of mice with sunitinib (25 mg/kg) began 4 hours prior to cell implantation and occurred twice daily. After 5 days, Matrigel plugs were removed and hemoglobin was quantified. Graph is representative of three independent experiments and indicates mean ± SEM from one experiment. Asterisks denote statistically significant (*, *p<*0.05; **p<0.01) differences compared to vehicle controls.

BMP7v was engineered to address poor PK issues that plague the wild type protein [16], and therefore represents a significant step forward in its potential use in treating cancer. Unlike wild type BMP7, BMP7v was able to avoid inhibition by circulating endogenous inhibitors such as noggin, chordin and chordin-like 2 via reduced binding (S1 Fig), and for all of these reasons we chose to continue to evaluate this molecule preclinically. Previous results indicated an anti-angiogenic activity of BMP7v in intracerebral GSLC xenografts, possibly contributing to its anti-tumor activity [16]. Therefore, we further characterized the anti-angiogenic activity of BMP7v. An *in vitro* co-culture system containing a feeder layer of ADSCs, which are similar to mesenchymal stem cells, and ECFCs, a subtype of umbilical cord blood-derived endothelial cells which can form vascular networks, was used to analyze BMP effects on cord formation [17,18]. Cords were visualized by immunostaining for the endothelial cell-specific marker “cluster of differentiation 31” (CD31) for ECFCs, and α-smooth muscle actin (SMA) for ADSCs. ADSCs migrate near and around developing cords and differentiate into pericyte-like cells similar to what is seen in normally developing vasculature [21]. BMP7, BMP7v, BMP9, and BMP10 significantly (*p<*0.001) reduced VEGF and bFGF-driven neo-angiogenesis (Fig 1A), while BMP4 only slightly enhanced cord formation compared to the PBS treated control (Fig 1A). BMP4, BMP7, BMP7v, BMP9, and BMP10 enhanced SMA staining, providing further support for their role in differentiation (with both BMP9 and BMP10 eliciting a profound response) (Fig 1A).

The anti-angiogenic effects of BMP7v observed *in vitro* prompted analysis of BMP7v effects on a neoangiogenesis Matrigel plug model that consists of the same ADSC/ECFC co-culture but can form functional blood vessels following co-implantation into the flank of a nude mouse [18,22]. Five days after implantation, the cells formed extensive networks of blood vessels whose functionality was assessed by measuring hemoglobin content. Both BMP7v (100 ng) and sunitinib (25 mg/kg) caused a significant reduction (*p<*0.05) in hemoglobin content (Fig 1D).

### BMP7v acts directly on endothelial cells to prevent VEGF and FGF-driven neo cord formation and regresses established cords

The ECFC/ADSC co-culture is a complex system requiring interactions of different cell types to form cords and the effects of the BMPs may be due to indirect effects mediated by the ADSCs. ADSCs and ECFCs were therefore individually assayed for response to BMPs using a simplified *in vitro* assay of culturing endothelial cells on basement membrane extract to form tubes [23]. HUVECs pre-treated for 48 or 72 hours with BMP7v prior to plating on Matrigel demonstrated a significant reduction (*p* < 0.001) in tube formation compared to PBS controls (Fig 1B) whereas BMP4 pre-treatment was largely ineffective. Additionally, mono-culture of HUVECs and ECFCs on tissue culture treated plastic were pre-treated with BMP7v and assayed for various angiogenic markers via qPCR and ELISA (S1 Table; all genes *p<*0.05). Many pro-angiogenic genes were down regulated at the mRNA and protein level further suggesting a direct effect of BMP7v on ECFCs.

**Fig 2:**
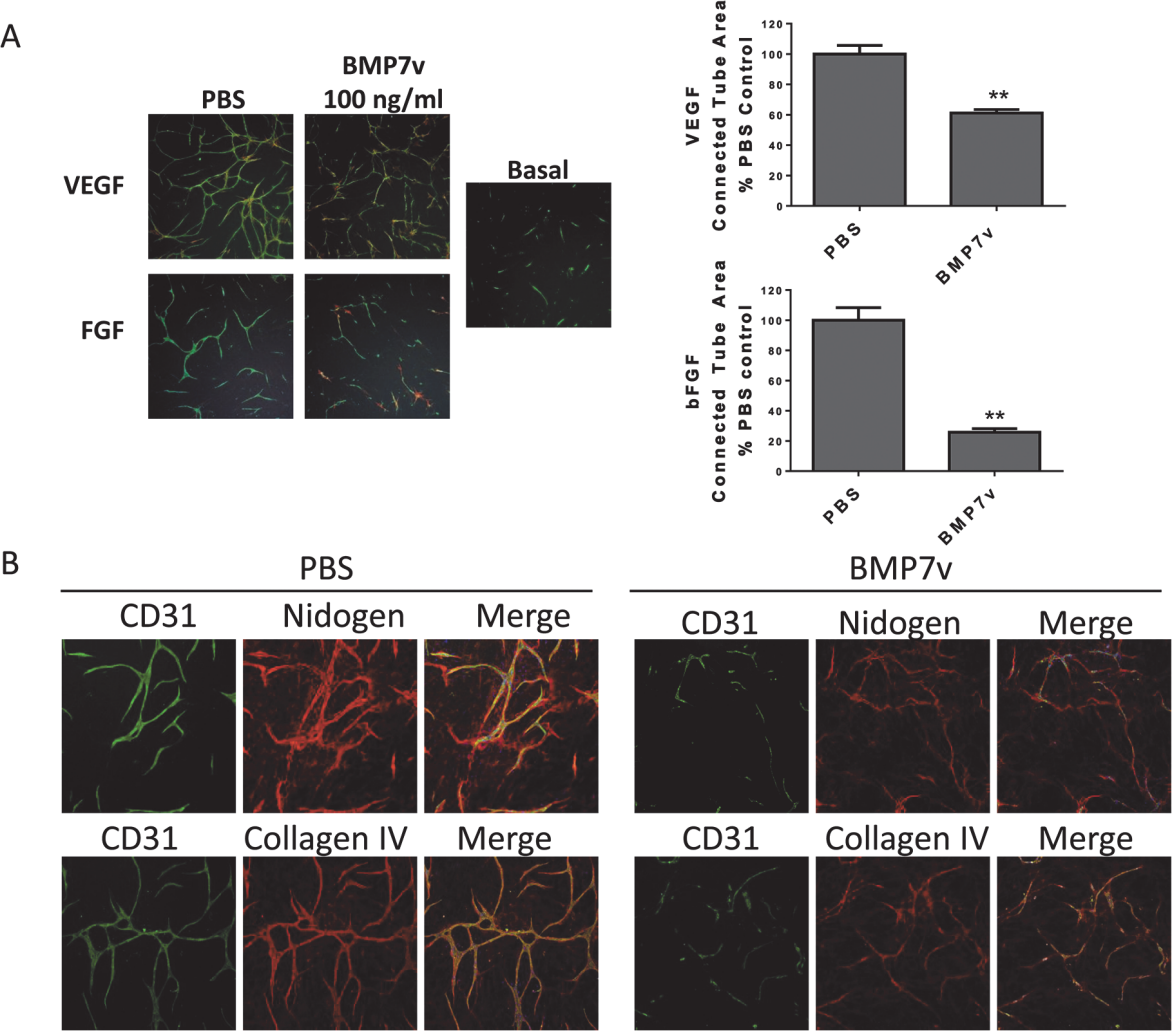
BMP7v reduced VEGF and bFGF established cords. (A) The ADSC/ECFC co-culture was unstimulated (basal) or stimulated with 10 ng/ml VEGF or bFGF for 96 hours prior to treatment with PBS or 100 ng/ml BMP7v for 72 hours and immunohistochemistry for CD31 (green), α-smooth muscle actin (red), and Hoechst 33342 to stain all nuclei (blue). Representative images (4X magnification) from three independent experiments are shown, and graphs represent mean connected tube area ± SEM after normalization to growth factor-induced cord values; asterisks denote statistically significant (**, *p<*0.01) differences compared to PBS controls. (B) The ADSC/ECFC co-culture was unstimulated (basal) or stimulated with 10 ng/ml bFGF for 96 hours prior to treatment with PBS or 100 ng/ml BMP7v for 72 hours and immunohistochemistry for CD31 (green), nidogen or collagen IV (red) and Hoechst 33342 to stain all nuclei (blue). Representative images (5X magnification) are shown.

To investigate the effects of BMP7v on ECFCs alone in the co-culture system, ECFCs were pre-treated with BMP7v *prior* to cord formation analysis. ECFCs pre-treated with BMP7v followed by removal of BMP7v prior to cord formation resulted in a significant reduction in growth factor-driven cord formation (*p<*0.001, Fig 1C). Pre-treatment of ECFCs along with subsequent *addition* of BMP7v led to the greatest reduction in cord formation (Fig 1C). Interestingly, cords that were established with growth factor prior to BMP7v treatment showed statistically significant regression (*p<*0.01, Fig 2) suggesting BMP7v can also disrupt formed vessels. VEGF-driven established cords were less sensitive to BMP7v than FGF-driven established cords, which responded with a complete breakdown in cord structure while maintaining nidogen immunostaining, a well-defined marker for basement membrane (Fig 2A and 2B) [24]. These data suggest that BMP7v has a direct effect on ECFCs and their angiogenic function, and can uniquely inhibit establishment of newly formed cords, as well as regression of firmly established cords.

### BMP7v does not alter proliferation or death of ECFCs but limits migration

An obvious potential anti-angiogenic mechanism of BMP7v involves regulation of ECFC growth, viability and/or migration. As determined through trypan blue exclusion, cell viability of the co-culture was not altered by BMP7v (data not shown) but migration of ECFCs alone was significantly, albeit weakly reduced (*p<*0.05, S2A Fig). ECFC proliferation (Ki67) and apoptosis (TUNEL) was not significantly affected by BMP7v (S2B Fig).

### BMP7v reduced tumor cell-driven endothelial cord formation

**Fig 3:**
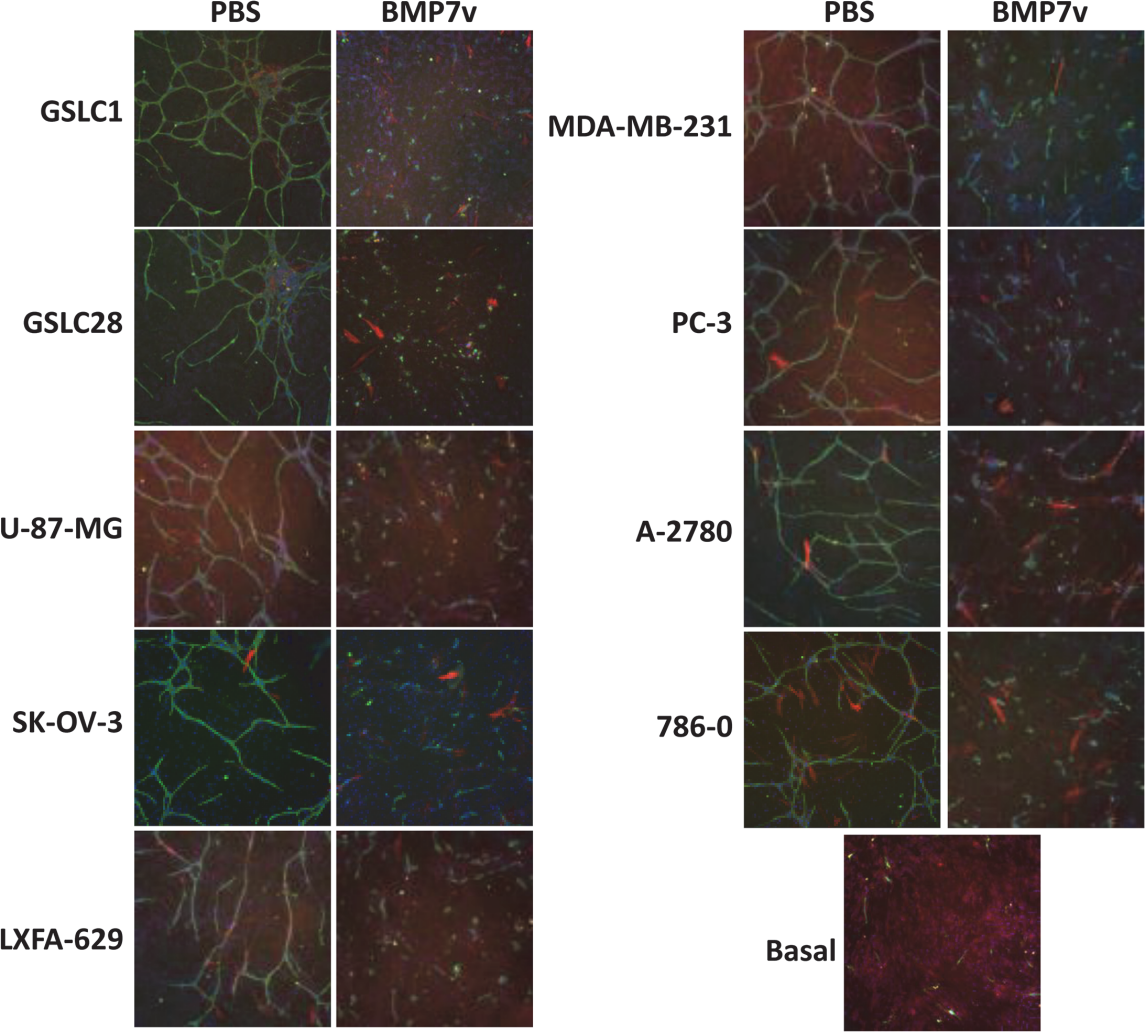
BMP7v reduced tumor-driven cord formation. ADSC/ECFC co-cultures with permeable transwells containing media (no cells-basal) or indicated tumor cells (GSLC1, GSLC28, A-2780, SK-OV-3, LXFA-629, MDA-MB-231, PC-3, U-87-MG, and 786–0) were treated with PBS or 100 ng/ml BMP7v for 96 hours prior to immunohistochemistry for CD31 (green), α-smooth muscle actin, (red), and Hoechst 33342 to stain all nuclei (blue). Representative images (5X magnification) are shown. All tumor cell lines treated with BMP7v displayed statistically significant (***, *p<*0.001) differences in connected tube area compared to PBS controls.

To more closely represent tumor angiogenesis, BMP7v effects on tumor cell-driven cord formation were analysed. Tumor cells from a range of histologies releasing soluble proangiogenic factors were grown in a permeable transwell system with a co-culture of ECFCs and ADSCs, followed by analysis of cord formation. BMP7v significantly reduced (*p<*0.001) cord formation induced by all tumor cell histologies tested, including GSLCs, suggesting a broad anti-angiogenic function of BMP7v *in vitro* (Fig 3).

### BMP7v activated SMAD signaling, down regulated MAPK family and AKT signaling, and reduced VEGFR2 and FGFR1 expression in endothelial cells

**Fig 4:**
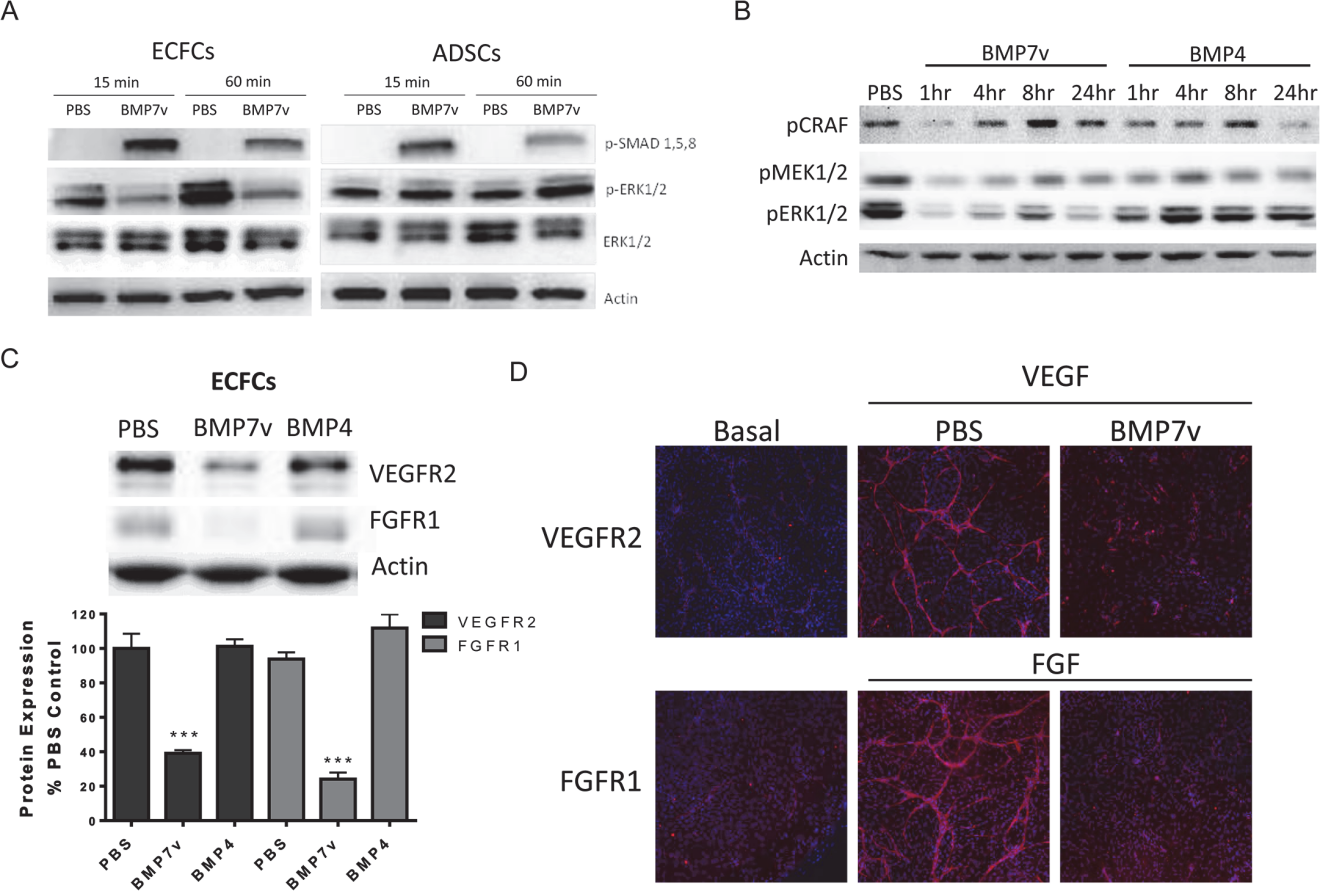
BMP7v reduced VEGFR2 and FGFR1 expression in endothelial cells. (A) Whole cell protein extracts were isolated following 15 or 60 minute PBS or 100 ng/ml BMP7v treatment and subjected to Western blot analysis using antiserum directed against phospho-SMAD1,5,8 (pSMAD1,5,8), phospho-ERK1/2 (pERK1/2), ERK1/2, and β-actin as a loading control. Results shown are representative of three independent experiments. (B) ECFCs were treated with PBS or 2nM BMP7v or BMP4 in ADSC conditioned defined co-culture media for the times indicated. Whole cell protein extracts were isolated following treatment and subjected to Western blot analysis using antiserum directed against phospho-CRAF (pCRAF), phospho-MEK1/2 (pMEK1/2), phospho-ERK1/2 (pERK1/2), and β-actin as a loading control. (C) Whole cell protein extracts were isolated from ECFCs following 72 hours of PBS or 100 ng/ml BMP7v treatment and subjected to Western blot analysis using antiserum directed against VEGFR2, FGFR1, and β-actin as a loading control. Graph represents mean densitometry ± SEM from three independent experiments, and asterisks denote statistically significant (***, *p<*0.001) differences compared to PBS controls. (D) The ADSC/ECFC co-culture was unstimulated (basal) or stimulated with 10 ng/ml VEGF or bFGF for 96 hours prior to treatment with PBS or 100 ng/ml BMP7v for 72 hours prior to immunohistochemistry for VEGFR2 (VEGF) or FGFR1 (bFGF) (red) and Hoechst 33342 to stain all nuclei (blue). Representative images (5X magnification) are shown. Upon BMP7v treatment, mean staining intensity for VEGFR2 and FGFR1 was statistically decreased (***, *p<*0.001) compared to PBS controls.

BMP7v activated the canonical SMAD signaling pathway in ECFCs and ADSCs as determined by SMAD phosphorylation (Fig 4A), which indicates functional BMP7 receptor expression. BMPs can also activate MAPK pathways; therefore, phosphorylation of p38 MAPK, ERK, and JNK was analysed. In ECFCs grown in ADSC conditioned media (since we could not directly assess this cell population in the context of the cord formation assay), BMP7v strongly *reduced* the level of pERK (Fig 4B). In addition, pAKT was significantly reduced (S3 Fig). The reduction was not a result of global signaling shutdown as p38 MAPK and SAPK/JNK signaling was largely unaffected (S3 Fig).

Therefore BMP7v was able to activate the canonical SMAD activation pathway while simultaneously and selectively down regulating signals emanating from growth (ERK) and survival (AKT) pathways. BMP7v strongly reduced protein expression of VEGFR2 and FGFR1 in ECFCs alone (Fig 4C) and in ECFCs (>80% reduction in protein expression) within the cord formation assay (Fig 4C and 4D). Previously, we reported on the shRNA knockdown of either VEGFR2 or FGFR1 in ECFCs leading to complete lack of response to either VEGF or FGF (18), respectively, and it is likely that BMP7v anti-angiogenic activity is at least partially a result of a SMAD-independent reduction in VEGFR2 and FGFR1 expression.

**Fig 5:**
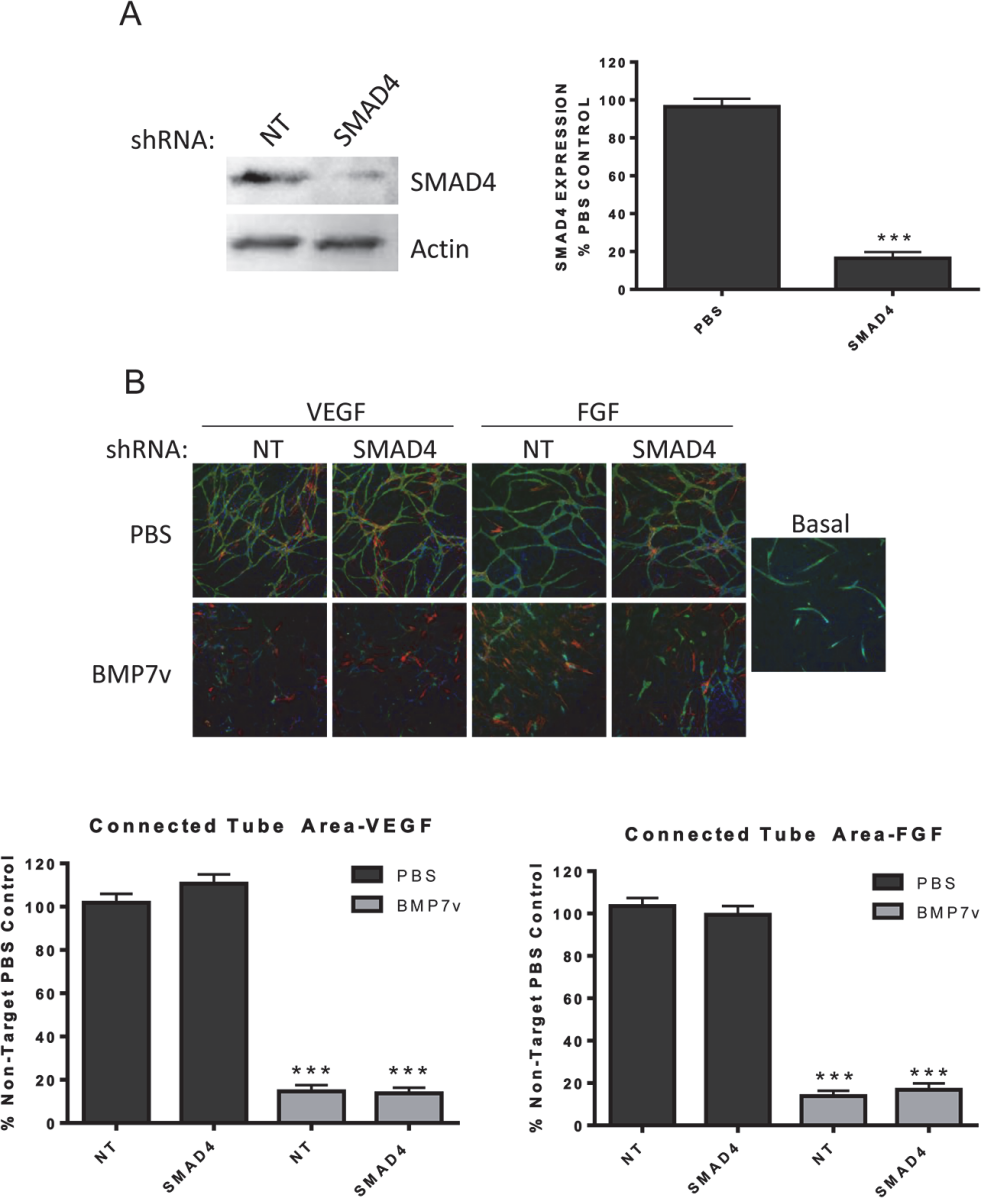
BMP7v inhibited cord formation in a SMAD4-independent manner. (A) Whole cell protein extracts were isolated from ECFCs following non-target (NT) or SMAD4 shRNA treatment and stable selection with puromycin. Extracts were subjected to Western blot analysis using antiserum directed against SMAD4 and β-actin as a loading control. Graph represents mean densitometry ± SEM from three independent experiments, and asterisks denote statistically significant (***, *p<*0.001) differences compared to PBS controls. (B) ECFCs were treated with non-target (NT) or SMAD4 shRNA followed by stable selection prior to plating into the ADSC/ECFC co-culture. The co-culture was stimulated with 10 ng/ml VEGF or bFGF simultaneously with PBS or 100 ng/ml BMP7v for 72 hours prior to immunohistochemistry for CD31 (green), α-smooth muscle actin (red), and Hoechst 33342 to stain all nuclei (blue). Representative images (5X magnification) are shown, graphs represent mean connected tube area ± SEM after basal cord formation data was subtracted from three independent experiments, and asterisks denote statistically significant (***, *p<*0.001) differences compared to PBS controls.

In order to determine if the anti-angiogenic effect of BMP7v is mediated through canonical SMAD signaling, stable shRNA knockdown in ECFCs of SMAD4, the obligatory nuclear shuttle protein for activated pSMAD1, 5, 8, was analysed for effects on cord formation. Surprisingly, shRNA knockdown of SMAD4 (>80% reduction in protein expression; Fig 5A) in ECFCs had little effect on VEGF and bFGF driven cord formation with or without BMP7v compared to shRNA knockdown of a non-targeting control (Fig 5B).

### BMP7v reduced angiogenesis in GSLC xenografts

**Fig 6:**
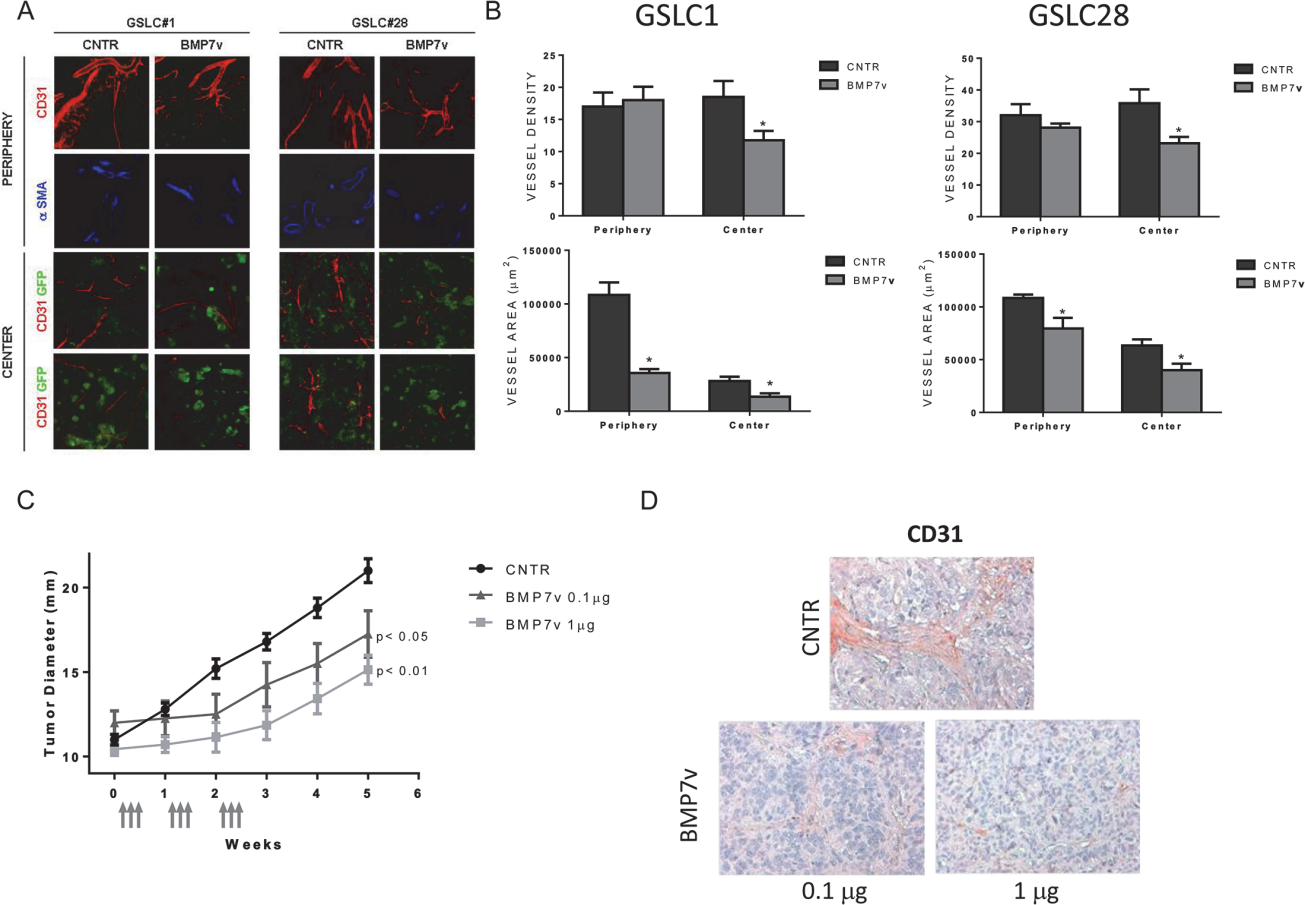
BMP7v reduced vascularization *in vivo*. (A) Assessment of vascularity with immunofluorescence microscopy in Matrigel implants containing either GSLC1 or GSLC28 GFP expressing tumor cells (green) from control (CNTR) or BMP7v treated mice. The peripheral and central regions of implants were assessed for CD31 (red) and α-SMA (blue). (B) Graphs represent mean vessel density (number of vessels per microscopic field), total vessel area, and relative vessel area (total vessel area/area of microscopic field) from one experiment, and asterisks denote statistically significant (*, *p<*0.05) differences compared to controls. (C) Mice developing subcutaneous xenografts in the range of 9–13 mm entered the treatment schedule (vehicle n, 5; BMP7v 1 μg, n, 7; BMP7v 0.1 μg, n, 4). Time point “0” corresponds to the beginning of treatment. Values are expressed as means ± SEM, and arrows depict compound treatment. BMP7v was well tolerated with no apparent loss of body weight or overt signs of toxicity. In vehicle injected mice, the tumors progressively increased their diameter from 9.9 + 0.74 mm (mean + SEM) at Week 1 to 21 + 1.6 mm at Week 5. Statistically significant (*p*<0.05) decreases in tumor size were observed at the 4 and 5 week measurements with 0.1 μg BMP7v treatment and (*p*<0.01) at the 2, 3, 4, and 5 week timepoints with 1 μg BMP7v compared to vehicle controls (CNTR). (D) Analysis of CD31 immunostaining from GSLC efficacy study tumors as described in Methods.

Subcutaneous grafting of GSLCs as Matrigel implants in immunosuppressed mice provides a well-suited model to study early tumor angiogenesis *in vivo [25]*. Four weeks after grafting, the implant appeared as a gelatinous mass with small vessels travelling across the surface (S4A Fig). The center of the implant was populated by clusters of tumor cells which strongly expressed nestin, a neuronal stem cell marker and, to a lesser degree, GFAP, an astrocyte differentiation marker (S4B Fig). Immunofluorescence of anti-CD31 revealed that the implant was crossed at its periphery by vessels arising from the host, which sprouted tiny branches towards the GFP-expressing tumor cell clusters (S4C Fig). These vessels showed a well-defined wall with endothelial cells packed in the typical cobblestone pattern with α-SMA staining cells, likely to be pericytes, attached (and denoting a relatively established vasculature as compared to the central structures). Conversely, the vascular channels that sprouted amongst the clusters of central tumor cells were irregular in shape and their walls often appeared discontinuous. Systemic administration of BMP7v resulted in a dose-dependent, tumor growth delay (p values <0.01 and <0.05 for 1 and 0.1 μg doses, respectively) similar to what was previously reported for orthotopic GSLC tumors [16]. Quantitative image analysis of the vessels revealed that BMP7v exerted an inhibitory effect on the vascularization of the implants (Fig 6A), which was more pronounced in the center (immature vessels) than the periphery (established vessels). In BMP7v treated implants, the number of neovessels in the center of both GSLC1 and GSLC28 implants was significantly reduced (*p<*0.05) compared with controls (S2 Table and Fig 6B). In the central region, both the total and relative vessel area was significantly lower (*p<*0.05) than controls (S2 Table and Fig 6B). Though there was not a significant difference in the number of peripheral vessels between control and BMP7v treated mice, their area did differ significantly (*p<*0.05) due to the smaller vessel surface following BMP7v treatment (S2 Table and Fig 6B). It is worth noting that in both control and BMP7v treated mice, the vessels at the implant periphery contained α-SMA expressing cells, which were absent in the newly formed vessels of the central region.

Control tumors displayed a typical histological pattern of GSLCs grown subcutaneously (S5 Fig). Prominent features included extensive areas of necrosis, arrangement of tumor cells in perinecrotic palisades, and remarkable angiogenesis. The tumor cells were rounded or polygonal in shape conferring an epithelial appearance to the tumor parenchyma. The proliferation index (Ki67 immunostaining) was 18–20%. Nestin was expressed at high levels by virtually all tumor cells while ~30–40% of the cells expressed GFAP and βIII tubulin was weakly present in ~5–8% of the cells (S6 Fig). In xenografts treated with BMP7v (1 μg), confluence of cystic spaces and reduction in angiogenesis were the most dramatic changes. Clusters of tumor cells with elongated morphology were surrounded by fibrous tissue in many regions of the tumor. Nestin was expressed in <50% of the tumor cells with GFAP expressed throughout. There was a dramatic increase in GFAP and βIII tubulin expression in tumor cells (40–50%), most of which displayed an elongated morphology with a concomitant reduction in Ki67 expression (4–5%). To determine if BMP7v inhibits the GSLC-derived angiogenesis, we measured the human MVD in BMP7v treated or control tumors. After confirming the species specificity of the antibodies (data not shown), BMP7v treatment clearly resulted in a statistically significant reduction in MVD following quantitation of human or both mouse and human CD31 (Fig 6D and S6 Fig).

## Discussion

In addition to its direct tumor cell effects in preventing the growth of GSLCs, our previous work on BMP7v suggested an anti-angiogenic role for this agent [16]. Here we show that BMP7v is an anti-angiogenic molecule *in vitro* and *in vivo* and acts directly on endothelial cells to mediate this effect. BMP7v is the only reported systemically available BMP in the context of cancer therapy and, when combined with a marked reduction in binding to endogenous BMP inhibitors such as chordin, chordin-like 2 and noggin represents a potentially significant advance in the field. BMP7v treatment of ECFC and HUVEC mono cultures induced multiple changes in gene and protein expression, in particular pro-angiogenesis molecules (including VEGFR2 and PlGF). In addition, when HUVECs were pretreated with BMP7v and cultured in Matrigel, there was a large reduction in the number of endothelial tubes formed. Furthermore, cord area was decreased by BMP7v when ECFCs were pretreated prior to co-culture with ADSCs. An examination of exogenous factors revealed that BMP7v inhibited cord formation in both VEGF and bFGF stimulated co-cultures, two most prominent stimulators of neovascularization [26,27]. Additionally, BMP7v inhibited tumor-driven cord formation across a range of human tumor cell models demonstrating that BMP7v is effective in a complex “physiological system.” Our previous results indicated that BMP7v did not alter expression of secreted proangiogenic factors from tumor cells [16], and BMP7v did not alter VEGF or bFGF secretion from the co-culture (data not shown). These data indicate that BMP7v may directly alter the ability of endothelial cells to form cords rather than affect the synthesis and/or release of soluble proangiogenic factors. In total, our *in vitro* data support a direct role for BMP7v modulating endothelial cell angiogenesis.

At least some of the anti-angiogenic effects of BMP7v may be explained by decreases in VEGFR2 and FGFR1 levels in ECFCs (60% and 80%, respectively). Given the large number of gene changes observed in the mono cultures, there are likely a host of additional genes exclusively affected by BMP7v, further contributing to the anti-angiogenic effect. For example, expression of proangiogenic signaling molecules such as cKIT and KIT ligand, as well as PlGF are significantly impaired by BMP7v treatment. The BMP7v-driven anti-angiogenic effect is in contrast to current anti-VEGF therapies that block a single growth factor pathway and therefore have a more focused and defined phenotypic effect that falls short of regressing established cords [28,29]. Unlike anti-VEGF therapies, BMP7v effectively prevented neovascularization and regressed growth factor-dependent established cords *in vitro*, though the latter effect was much stronger for FGF-dependent cords than VEGF-dependent ones. This raises the intriguing possibility that BMP7v may be an effective anti-angiogenic agent in tumors that shift away from VEGF-dependent angiogenesis to a more FGF-dependent state [30].

To understand the more detailed mechanism by which BMP7v affected vessels, we investigated a number of additional BMP-dependent functions. Upon binding type I and type II receptor homodimers, BMPs activate SMADs 1, 5, and 8, which then associate with SMAD4, leading to nuclear translocation and gene expression [31]. While BMP7v potently stimulated phosphorylation of SMADs 1, 5, and 8 in ECFCs, significant knockdown of SMAD4 still resulted in inhibition of cord formation. Therefore, either SMAD signaling was not required for the anti-angiogenic effect of BMP7v, or a very small amount of SMAD4 expression was sufficient to mediate signal. It is known that BMPs can exert their actions via non-SMAD pathways [2,3]. For example, BMP7 stimulation of chemotaxis of monocytic cells occurred in a SMAD4-independent fashion [32]. Another pathway crucial to angiogenesis, RAS/ERK [33], was inhibited by BMP7v in growth factor stimulated ECFCs and it is reported that ERK and SMAD pathways can work together to regulate angiogenesis [33,34]. In fact there are numerous reports of a positive role for the ERK pathway in directly controlling angiogenesis, strengthening the likelihood that BMP7v down regulation of ERK activity, coupled with a significant reduction in proangiogenic receptor expression, significantly contributed to its antiangiogenic response [35,36]. Activation of the SMAD pathway and down-regulation of ERK pathways by BMP7v may therefore be critical in inducing an anti-angiogenic phenotype. Furthermore, there are additional signaling proteins that are modulated by BMPs, including p38 MAPK, JNK, STATs, and PI3K/mTOR, but these pathways appear to be largely unaffected by BMP7v treatment [37–40].

Clearly, BMP7v did not block *in vitro* angiogenesis via an apoptotic or antiproliferative response. While there was a slight reduction in ECFC migration upon BMP7v treatment, it is unlikely that this effect is responsible for the potent anti-angiogenic effect. Despite the lack of overt apoptotic or anti-proliferative markers, cords *in vitro* were clearly eliminated following BMP7v treatment, as evidenced through persistent nidogen and collagen IV immunostaining (reflecting the maintenance of a “basement membrane”[41]) in the absence of coincident CD31 immunostaining. One possibility is that BMP7v caused the ECFCs to differentiate into a cell type incapable of supporting cord formation (endoMT) [42] much like BMP4 which was shown to convert endothelial cells into stem-like cells [43]. Our attempts to demonstrate that BMP7v induced a similar differentiation event in ECFCs were unsuccessful (data not shown). An additional possibility is that BMP7v may have induced programmed necrosis in endothelial cells, a potentially important mechanism for tissue remodeling [44].

Importantly, BMP7v inhibited vessel formation in two different *in vivo* models; in ADSC/ECFC Matrigel plugs as measured by hemoglobin content and in a GSLC xenograft as evidenced by a reduction in neo-formed host vessel area and number. We previously reported that BMP7v in part exerted its anti-tumoral effect on GSLC tumors through an inhibition of angiogenesis [16] and our current results support this hypothesis. Interestingly, the effect of BMP7v was strongest within the tumor center, where vascularization was irregular and α-SMA-staining pericytes were not found, compared to vessels in the periphery which clearly formed more established, pericyte-associated structures [28,45]. Given that BMP7v down regulated VEGFR2 and FGFR1, and that VEGF and bFGF are key players in new vessel formation, it is not surprising that the immature, central vessels would be more sensitive to BMP7v. While BMP7v did not affect vessel *number* in the periphery, there was a significant change in vessel *area*. Therefore, BMP7v did have an effect on the more established peripheral vessels and raises the intriguing possibility that BMP7v is inducing what Jain described as “vascular normalization”, as opposed to the outright regression seen in the implant center [46].

BMP7v administered at or above doses of 0.1 μg reduced the growth rate of GSLC xenografts. This effect depended on a continuous supply of BMP7v because tumor size increased upon BMP7v discontinuation. However, the apparent tumor regrowth that followed discontinuation of treatment may partly be ascribed to the development of cysts and not entirely to tumor regrowth. The development of cysts was associated with the occurrence of fibrous septa and sclerotic areas in the context of the tumor parenchyma, strongly suggesting that regression had occurred. The occurrence of regressive changes was paralleled by a reduction in the density of microvessels and by astrocytic and neuronal differentiation. Therefore, intraperitoneal administration of BMP7v inhibited the growth of subcutaneous GSLC xenografts and resulted from a reduction in cell proliferation, occurrence of regressive phenomena, inhibition of tumor angiogenesis and enhancement of neural cell differentiation.

In summary, BMP7v sets into motion changes in endothelial cell function that ultimately lead to a reduction in organized vessels and endothelial cell markers. In addition, BMP7v has been shown to play a role in promoting cancer stem cell differentiation as well as a significant reduction in GSLC growth *in vivo* [16]. Therefore, a dual effect of BMP7v on both tumor stem cell differentiation and the invading endothelium may result in a potentially novel anti-tumor therapy.

## Supporting Information

S1 Fig Analysis of wild-type BMP7 and BMP7v binding inhibitors. Hep3B2 cells stably transfected with a hepcidin promoter luciferase construct (Hep3B2_HepPro_luc) were used for BMP inhibitor experiments. Hep3B2_HepPro_luc cells were plated at 30,000 cells per well in a tissue culture treated 96 well plate in DMEM (Hyclone) 5% FBS (Gibco) supplemented with Non-essential amino acids (Hyclone) and 200 μg/ml gentecin (Hyclone) for 24 hours. Cells were then starved in OMEM + 0.2% BSA for 5 hours. Cells were treated with a mixture of BMP and inhibitors in OMEM (Gibco) + 0.2%BSA (Gibco) for 18 hours then developed for luciferase activity utilizing Luciferase reporter Gene Assay Kit (Roche). BMPs were added at concentrations in the linear range of the assay (BMP-7s were used at 1 nM and 100 nm. Inhibitors were added at 100X molar excess of the BMP. BMP-7s were generated at Eli Lilly and Company.
(TIF)

S2 Fig BMP7v reduced endothelial cell migration. (A) ECFCs were plated into Oris cell migration plates with the stopper in place. Following 24 hours, stoppers were removed and PBS or 1.6 nM BMP7v or BMP4 was added and cells were allowed to migrate for 24 hours prior to fixation and staining with propidium iodide (scratch area image represents stopper in place for entire experiment just prior to fixation). Cells were imaged on the Acumen Explorer and cell number within the scratch area (green box; blue box designates non-scratch area) was analysed. Representative images are shown, numbers represent percent decrease in cell number in the scratch area compared to the PBS control ± SEM from three independent experiments, and asterisks denote statistically significant (*, *p<*0.05) differences compared to PBS controls. (B) The ADSC/ECFC co-culture was stimulated with 10 ng/ml VEGF or bFGF simultaneously with PBS or 100 ng/ml BMP7v for 72 hours prior to immunohistochemistry for Ki67 or TUNEL (red) and Hoechst 33342 to stain all nuclei (blue). Representative images (5X magnification) are shown, graphs represent mean percent responders ± SEM from three independent experiments.
(TIF)

S3 Fig Effect of BMP7v on various signalling pathways. ECFCs were treated with PBS or 2nM BMP7v or BMP4 in ADSC conditioned defined co-culture media for the times indicated. Whole cell protein extracts were isolated following treatment and subjected to Western blot analysis using antiserum directed against phospho-CRAF (pCRAF), phospho-MEK1/2 (pMEK1/2), phospho-p38 MAPK (p-p38), pERK1/2 (pERK1/2), phospho-SAPK/JNK (pJNK), and β-actin as a loading control. Note, for the convenience of the reader, some of the blots of Fig 4 are repeated in this figure.
(TIF)

S4 Fig BMP7v reduced angiogenesis in GSLC xenografts (A) Appearance of the Matrigel implant upon microsurgical inspection at 4 weeks post grafting. (B) Histological pattern of Matrigel implant at low (a, H&E; scale bar, 2,500 μm) and higher magnification with details of peripheral (b) and central regions of implant (c) (H&E; scale bars, 80 μm). Anti-nestin immunostaining (d; scale bar, 30 μm). Fluorescence microscopy showing clusters of GFP positive GSLCs and their GFAP expression (GFAP *red* in e; scale bar, 30 μm). (C) Quantitative assessment of vascularity in Matrigel implants after anti-CD31 immunostaining (*red*, anti-CD-31; *green*, GFP; *blue*, DAPI) at the periphery (*upper panel*; scale bar, 150 μm) and center of implant (*lower panel*; scale bar, 80 μm).
(TIF)

S5 Fig BMP7v reduced proliferation and induced differentiation of GSLCs *in vivo*. Three weeks after the last treatment, control tumors (CNTR) or tumors treated with BMP7v (1 μg or 0.1 μg) were assessed for morphology by H&E staining. In addition, tumor cell proliferation was analysed by Ki67, and tumor cell differentiation was assessed by nestin, GFAP, and βIII tubulin (βIII Tub) immunohistochemistry.
(TIF)

S6 Fig BMP7v treatment reduced microvessel density (MVD) in tumor xenografts. (A) Immunohistochemistry was performed on vehicle (CNTR) or BMP7v treated (0.1 μg or 1 μg) tumors three weeks following treatment using a human-specific CD31 antibody. (B) Graph represents MVD assessed from both human and mouse CD31 immunostaining, and asterisks denote statistically significant (**, p<0.05; ***, p<0.001) differences compared to vehicle controls (CNTR).
(TIF)

S1 Table Summary of fold-change in gene expression upon BMP7v treatment. Quantitative PCR analysis was performed, and data was normalized to the geometric mean of at least three housekeeping genes. ND, not determined. Changes in red denote *p<*0.05.
(DOCX)

S2 Table Summary of results from quantitative assessment of angiogenesis in Matrigel implants. (DOCX)

## References

[1] Lowery JW, de Caestecker MP (2010) BMP signaling in vascular development and disease. Cytokine Growth Factor Rev 21: 287–298. 10.1016/j.cytogfr.2010.06.001 20674464 PMC2939258

[2] Sieber C, Kopf J, Hiepen C, Knaus P (2009) Recent advances in BMP receptor signaling. Cytokine Growth Factor Rev 20: 343–355. 10.1016/j.cytogfr.2009.10.007 19897402

[3] Wagner DO, Sieber C, Bhushan R, Borgermann JH, Graf D, et al (2010) BMPs: from bone to body morphogenetic proteins. Sci Signal 3: mr1 10.1126/scisignal.3107mr1 20124549

[4] Lee J, Jin Son M, Woolard K, Donin N, Li A, et al (2008) Epigenetic-mediated dysfunction of the bone morphogenetic protein developmental pathway inhibits differentiation of human glioblastoma tumor initiating cells. Cancer Cell 13: 69–80. 10.1016/j.ccr.2007.12.005 18167341 PMC2835498

[5] Piccirillo S, Reynolds B, Zanetti N, Lamorte G, Binda E, et al (2006) Bone morphogenetic proteins inhibit the tumorigenic potential of human brain tumour-initiating cells. Nature 444: 761–765. 17151667 10.1038/nature05349

[6] Piccirillo S, Vescovi A (2007) Bone morphogenetic proteins regulate tumorigenicity in human glioblastoma stem cells. Ernst Schering Foundation Symposium Proceedings 5: 59–81. 10.1007/2789_2007_044 17939295

[7] Silinsky J, Grimes C, Driscoll T, Green H, Cordova J, et al (2013) CD 133+ and CXCR4+ colon cancer cells as a marker for lymph node metastasis. J Surg Res 185: 113–118. 10.1016/j.jss.2013.05.049 23777983

[8] D'Amico L, Patane S, Grange C, Bussolati B, Isella C, et al (2013) Primary breast cancer stem-like cells metastasise to bone, switch phenotype and acquire a bone tropism signature. Br J Cancer 108: 2525–2536. 10.1038/bjc.2013.271 23801032 PMC3694250

[9] Reya T, Morrison SJ, Clarke MF, Weissman IL (2001) Stem cells, cancer, and cancer stem cells. Nature 414: 105–111. 11689955 10.1038/35102167

[10] Ricci-Vitiani L, Pallini R, Biffoni M, Todaro M, Invernici G, et al (2010) Tumour vascularization via endothelial differntiation of glioblastoma stem-like cells. Nature 468. 10.1038/nature09557 21102434

[11] Cunha SI, Pietras K (2011) ALK1 as an emerging target for antiangiogenic therapy of cancer. Blood 117: 6999–7006. 10.1182/blood-2011-01-330142 21467543 PMC3143549

[12] Levet S, Ciais D, Merdzhanova G, Mallet C, Zimmers TA, et al (2013) Bone morphogenetic protein 9 (BMP9) controls lymphatic vessel maturation and valve formation. Blood 122: 598–607. 10.1182/blood-2012-12-472142 23741013 PMC3724195

[13] Heinke J, Wehofsits L, Zhou Q, Zoeller C, Baar KM, et al (2008) BMPER is an endothelial cell regulator and controls bone morphogenetic protein-4-dependent angiogenesis. Circ Res 103: 804–812. 10.1161/CIRCRESAHA.108.178434 18787191

[14] Zhou Q, Heinke J, Vargas A, Winnik S, Krauss T, et al (2007) ERK signaling is a central regulator for BMP-4 dependent capillary sprouting. Cardiovasc Res 76: 390–399. 17850776 10.1016/j.cardiores.2007.08.003

[15] Rothhammer T, Wild PJ, Meyer S, Bataille F, Pauer A, et al (2007) Bone morphogenetic protein 7 (BMP7) expression is a potential novel prognostic marker for recurrence in patients with primary melanoma. Cancer Biomark 3: 111–117. 17522432 10.3233/cbm-2007-3205

[16] Tate C, Pallini R, Ricci-Vitiani L, Dowless M, Shiyanova T, et al (2012) A BMP7 variant inhibits the tumorigenic potential of glioblastoma stem-like cells. Cell Death and Differentiation 19: 1644–1654. 10.1038/cdd.2012.44 22539003 PMC3438495

[17] Meier T, Uhlik M, Chintharlapalli S, Dowless M, Van Horn R, et al (2011) Tasisulam sodium, an antitumor agent that inhibits mitotic progression and induces vascular normalization. Mol Cancer Ther 10: 2168–2178. 10.1158/1535-7163.MCT-11-0323 21903607

[18] Blosser W, Vakana E, Wyss LV, Swearingen ML, Stewart J, et al (2014) A method to assess target gene involvement in angiogenesis in vitro and in vivo using lentiviral vectors expressing shRNA. PLoS One 9: e96036 10.1371/journal.pone.0096036 24759702 PMC3997504

[19] Gough W, Hulkower KI, Lynch R, McGlynn P, Uhlik M, et al (2011) A quantitative, facile, and high-throughput image-based cell migration method is a robust alternative to the scratch assay. J Biomol Screen 16: 155–163. 10.1177/1087057110393340 21297103

[20] Pallini R, Ricci-Vitiani L, Banna G, Signore M, Lombardi D, et al (2008) Cancer stem cell analysis and clinical outcome in patients with glioblastoma multiforme. Clinical Cancer Research 14: 8205–7212. 10.1158/1078-0432.CCR-08-0644 19088037

[21] Falcon BL, Stewart J, Ezell S, Hanson J, Wijsman J, et al (2013) High-content multiplexed tissue imaging and quantification for cancer drug discovery. Drug Discov Today 18: 510–522. 10.1016/j.drudis.2012.08.008 22944609

[22] Traktuev DO, Merfeld-Clauss S, Li J, Kolonin M, Arap W, et al (2008) A population of multipotent CD34-positive adipose stromal cells share pericyte and mesenchymal surface markers, reside in a periendothelial location, and stabilize endothelial networks. Circ Res 102: 77–85. 17967785 10.1161/CIRCRESAHA.107.159475

[23] Arnaoutova I, George J, Kleinman HK, Benton G (2009) The endothelial cell tube formation assay on basement membrane turns 20: state of the science and the art. Angiogenesis 12: 267–274. 10.1007/s10456-009-9146-4 19399631

[24] Miosge N, Sasaki T, Timpl R (2002) Evidence of nidogen-2 compensation for nidogen-1 deficiency in transgenic mice. Matrix Biol 21: 611–621. 12475645 10.1016/s0945-053x(02)00070-7

[25] Falchetti ML, Mongiardi MP, Fiorenzo P, Petrucci G, Pierconti F, et al (2008) Inhibition of telomerase in the endothelial cells disrupts tumor angiogenesis in glioblastoma xenografts. Int J Cancer 122: 1236–1242. 18027853 10.1002/ijc.23193

[26] Adams RH, Alitalo K (2007) Molecular regulation of angiogenesis and lymphangiogenesis. Nat Rev Mol Cell Biol 8: 464–478. 17522591 10.1038/nrm2183

[27] Dow JK, deVere White RW (2000) Fibroblast growth factor 2: its structure and property, paracrine function, tumor angiogenesis, and prostate-related mitogenic and oncogenic functions. Urology 55: 800–806. 10840080 10.1016/s0090-4295(00)00457-x

[28] Nagy JA, Dvorak HF (2012) Heterogeneity of the tumor vasculature: the need for new tumor blood vessel type-specific targets. Clin Exp Metastasis 29: 657–662. 10.1007/s10585-012-9500-6 22692562 PMC3484269

[29] Sitohy B, Nagy JA, Jaminet SC, Dvorak HF (2011) Tumor-surrogate blood vessel subtypes exhibit differential susceptibility to anti-VEGF therapy. Cancer Res 71: 7021–7028. 10.1158/0008-5472.CAN-11-1693 21937680 PMC3217088

[30] Lieu C, Heymach J, Overman M, Tran H, Kopetz S (2011) Beyond VEGF: inhibition of the fibroblast growth factor pathway and antiangiogenesis. Clin Cancer Res 17: 6130–6139. 10.1158/1078-0432.CCR-11-0659 21953501 PMC5562355

[31] Heldin CH, Miyazono K, ten Dijke P (1997) TGF-beta signalling from cell membrane to nucleus through SMAD proteins. Nature 390: 465–471. 9393997 10.1038/37284

[32] Perron JC, Dodd J (2009) ActRIIA and BMPRII Type II BMP receptor subunits selectively required for Smad4-independent BMP7-evoked chemotaxis. PLoS One 4: e8198 10.1371/journal.pone.0008198 20011660 PMC2788225

[33] Rush S, Khan G, Bamisaiye A, Bidwell P, Leaver HA, et al (2007) c-jun amino-terminal kinase and mitogen activated protein kinase 1/2 mediate hepatocyte growth factor-induced migration of brain endothelial cells. Exp Cell Res 313: 121–132. 17055484 10.1016/j.yexcr.2006.09.018

[34] Eliceiri BP, Klemke R, Stromblad S, Cheresh DA (1998) Integrin alphavbeta3 requirement for sustained mitogen-activated protein kinase activity during angiogenesis. J Cell Biol 140: 1255–1263. 9490736 10.1083/jcb.140.5.1255 PMC2132684

[35] Sobczak I, Galabova-Kovacs G, Sadzak I, Kren A, Christofori G, et al (2008) B-Raf is required for ERK activation and tumor progression in a mouse model of pancreatic beta-cell carcinogenesis. Oncogene 27: 4779–4787. 10.1038/onc.2008.128 18490924

[36] Wimmer R, Cseh B, Maier B, Scherrer K, Baccarini M (2012) Angiogenic sprouting requires the fine tuning of endothelial cell cohesion by the Raf-1/Rok-alpha complex. Dev Cell 22: 158–171. 10.1016/j.devcel.2011.11.012 22209329 PMC3268451

[37] Tokuda H, Hatakeyama D, Shibata T, Akamatsu S, Oiso Y, et al (2003) p38 MAP kinase regulates BMP-4-stimulated VEGF synthesis via p70 S6 kinase in osteoblasts. Am J Physiol Endocrinol Metab 284: E1202–1209. 12637256 10.1152/ajpendo.00300.2002

[38] Rajan P, Panchision DM, Newell LF, McKay RD (2003) BMPs signal alternately through a SMAD or FRAP-STAT pathway to regulate fate choice in CNS stem cells. J Cell Biol 161: 911–921. 12796477 10.1083/jcb.200211021 PMC2172962

[39] Langenfeld EM, Kong Y, Langenfeld J (2005) Bone morphogenetic protein-2-induced transformation involves the activation of mammalian target of rapamycin. Mol Cancer Res 3: 679–684. 16380505 10.1158/1541-7786.MCR-05-0124

[40] Townsend KL, Suzuki R, Huang TL, Jing E, Schulz TJ, et al (2012) Bone morphogenetic protein 7 (BMP7) reverses obesity and regulates appetite through a central mTOR pathway. FASEB J 26: 2187–2196. 10.1096/fj.11-199067 22331196 PMC3336788

[41] Miosge N, Holzhausen S, Zelent C, Sprysch P, Herken R (2001) Nidogen-1 and nidogen-2 are found in basement membranes during human embryonic development. Histochem J 33: 523–530. 12005023 10.1023/a:1014995523521

[42] Catalano V, Turdo A, Di Franco S, Dieli F, Todaro M, et al (2013) Tumor and its microenvironment: a synergistic interplay. Semin Cancer Biol 23: 522–532. 10.1016/j.semcancer.2013.08.007 24012661

[43] Medici D, Shore EM, Lounev VY, Kaplan FS, Kalluri R, et al (2010) Conversion of vascular endothelial cells into multipotent stem-like cells. Nat Med 16: 1400–1406. 10.1038/nm.2252 21102460 PMC3209716

[44] Kreuzaler P, Watson CJ (2012) Killing a cancer: what are the alternatives? Nat Rev Cancer 12: 411–424. 10.1038/nrc3264 22576162

[45] Nagy JA, Chang SH, Shih SC, Dvorak AM, Dvorak HF (2010) Heterogeneity of the tumor vasculature. Semin Thromb Hemost 36: 321–331. 10.1055/s-0030-1253454 20490982 PMC3278036

[46] Jain RK (2005) Normalization of tumor vasculature: an emerging concept in antiangiogenic therapy. Science 307: 58–62. 15637262 10.1126/science.1104819

