# Supplementary figures and images for: A BMP7 Variant Inhibits Tumor Angiogenesis In Vitro and In Vivo through Direct Modulation of Endothelial Cell Biology
Source: PLoS One. 2015 Apr 28;10(4):e0125697. doi: 10.1371/journal.pone.0125697 (PMC4412825; doi:10.1371/journal.pone.0125697)

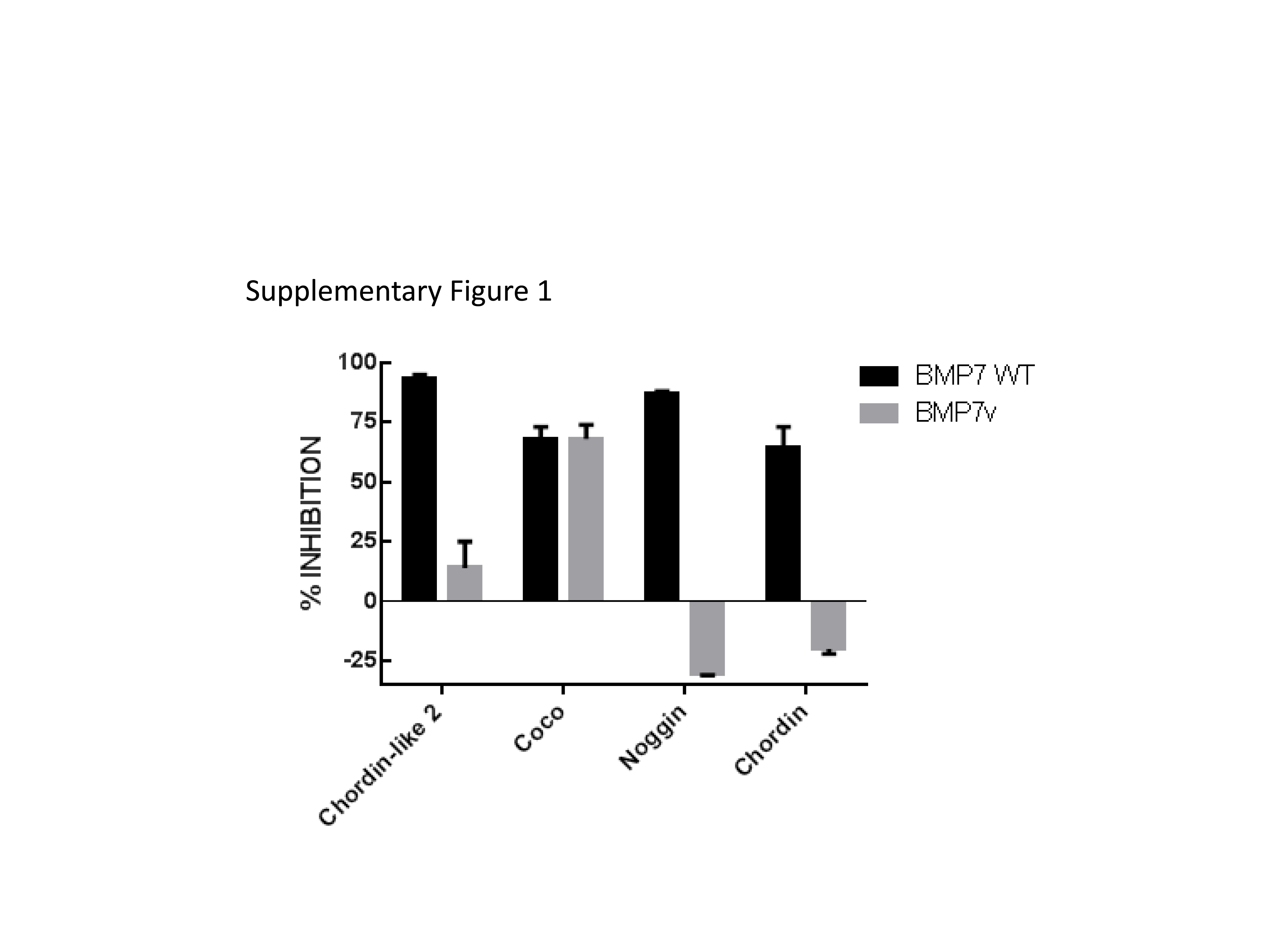

Supplement: S1 Fig — Hep3B2 cells stably transfected with a hepcidin promoter luciferase construct (Hep3B2_HepPro_luc) were used for BMP inhibitor experiments. Hep3B2_HepPro_luc cells were plated at 30,000 cells per well in a tissue culture treated 96 well plate in DMEM (Hyclone) 5% FBS (Gibco) supplemented with Non-essential amino acids (Hyclone) and 200 μg/ml gentecin (Hyclone) for 24 hours. Cells were then starved in OMEM + 0.2% BSA for 5 hours. Cells were treated with a mixture of BMP and inhibitors in OMEM (Gibco) + 0.2%BSA (Gibco) for 18 hours then developed for luciferase activity utilizing Luciferase reporter Gene Assay Kit (Roche). BMPs were added at concentrations in the linear range of the assay (BMP-7s were used at 1 nM and 100 nm. Inhibitors were added at 100X molar excess of the BMP. BMP-7s were generated at Eli Lilly and Company. (TIF) [file pone.0125697.s001.tif]

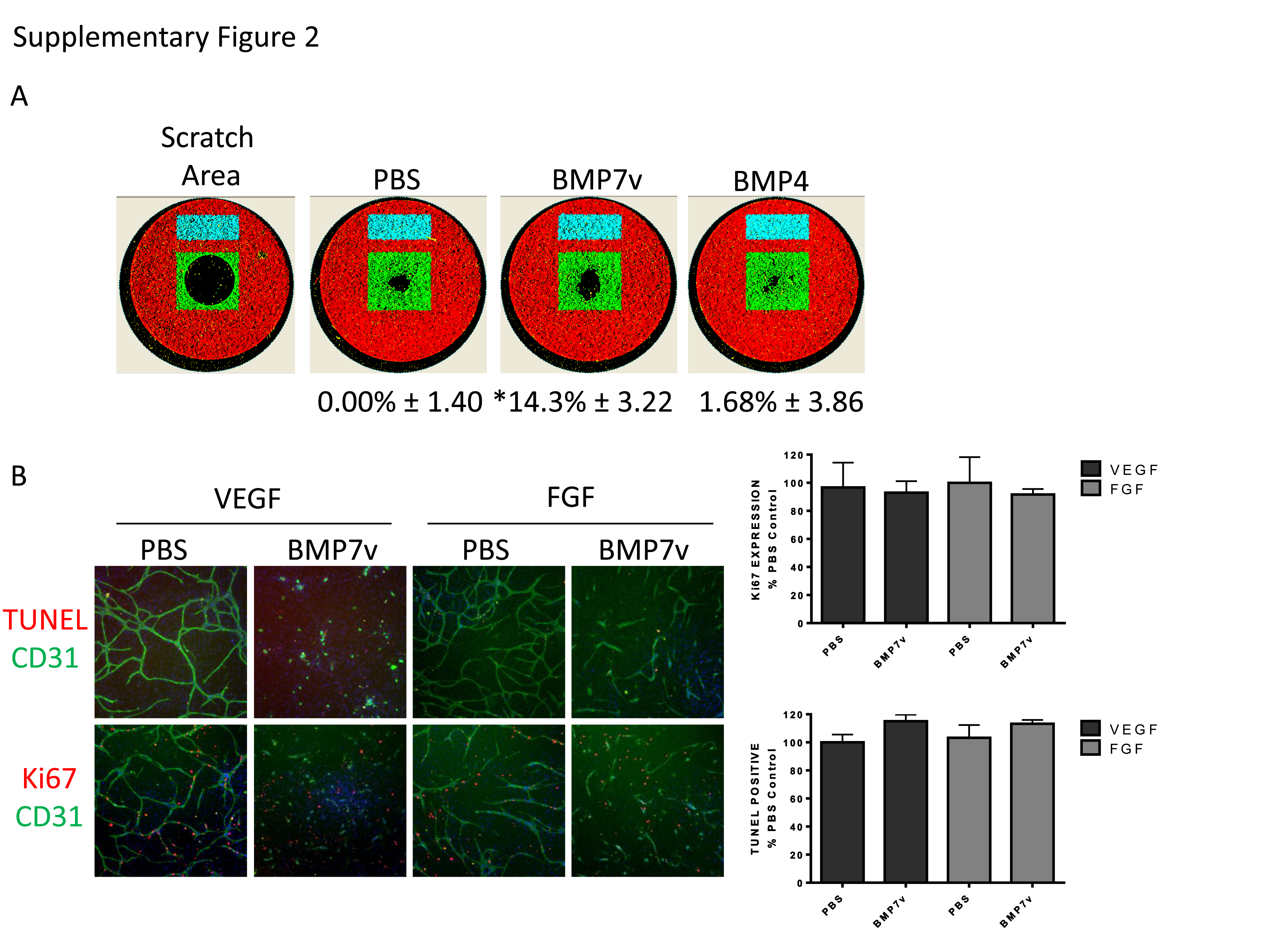

Supplement: S2 Fig — (A) ECFCs were plated into Oris cell migration plates with the stopper in place. Following 24 hours, stoppers were removed and PBS or 1.6 nM BMP7v or BMP4 was added and cells were allowed to migrate for 24 hours prior to fixation and staining with propidium iodide (scratch area image represents stopper in place for entire experiment just prior to fixation). Cells were imaged on the Acumen Explorer and cell number within the scratch area (green box; blue box designates non-scratch area) was analysed. Representative images are shown, numbers represent percent decrease in cell number in the scratch area compared to the PBS control ± SEM from three independent experiments, and asterisks denote statistically significant (*, p<0.05) differences compared to PBS controls. (B) The ADSC/ECFC co-culture was stimulated with 10 ng/ml VEGF or bFGF simultaneously with PBS or 100 ng/ml BMP7v for 72 hours prior to immunohistochemistry for Ki67 or TUNEL (red) and Hoechst 33342 to stain all nuclei (blue). Representative images (5X magnification) are shown, graphs represent mean percent responders ± SEM from three independent experiments. (TIF) [file pone.0125697.s002.tif]

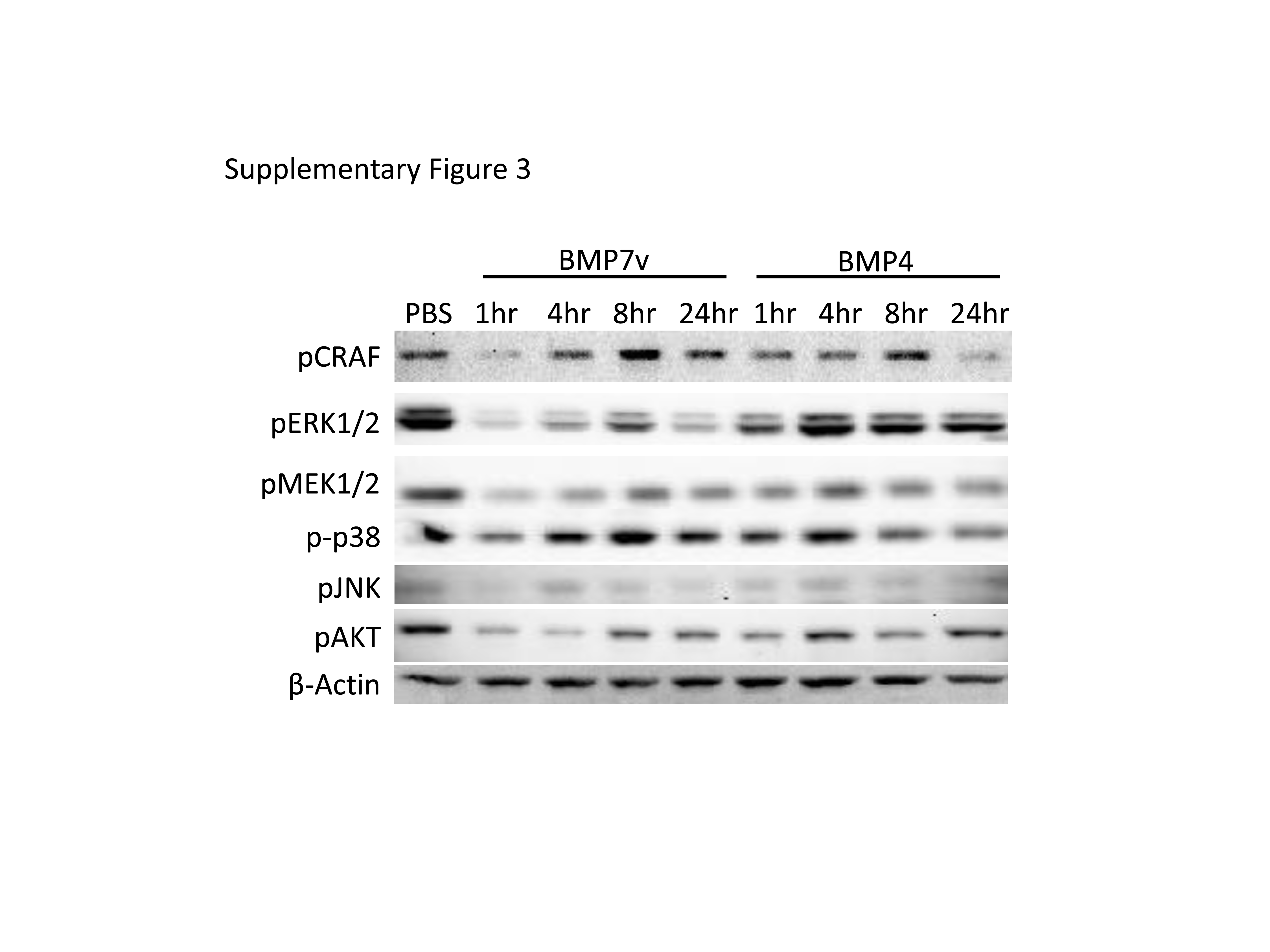

Supplement: S3 Fig — ECFCs were treated with PBS or 2nM BMP7v or BMP4 in ADSC conditioned defined co-culture media for the times indicated. Whole cell protein extracts were isolated following treatment and subjected to Western blot analysis using antiserum directed against phospho-CRAF (pCRAF), phospho-MEK1/2 (pMEK1/2), phospho-p38 MAPK (p-p38), pERK1/2 (pERK1/2), phospho-SAPK/JNK (pJNK), and β-actin as a loading control. Note, for the convenience of the reader, some of the blots of Fig 4 are repeated in this figure. (TIF) [file pone.0125697.s003.tif]

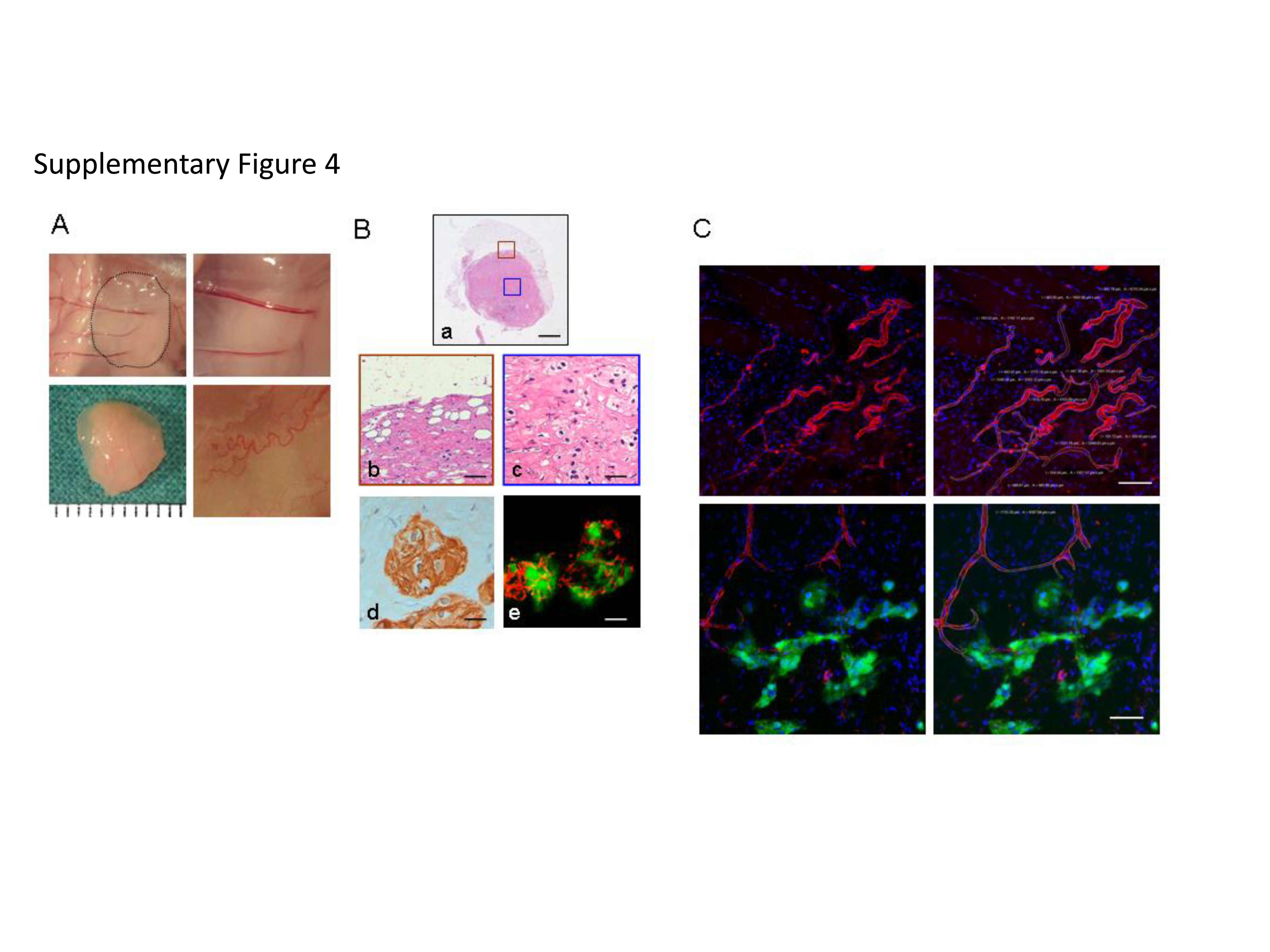

Supplement: S4 Fig — (A) Appearance of the Matrigel implant upon microsurgical inspection at 4 weeks post grafting. (B) Histological pattern of Matrigel implant at low (a, H&E; scale bar, 2,500 μm) and higher magnification with details of peripheral (b) and central regions of implant (c) (H&E; scale bars, 80 μm). Anti-nestin immunostaining (d; scale bar, 30 μm). Fluorescence microscopy showing clusters of GFP positive GSLCs and their GFAP expression (GFAP red in e; scale bar, 30 μm). (C) Quantitative assessment of vascularity in Matrigel implants after anti-CD31 immunostaining (red, anti-CD-31; green, GFP; blue, DAPI) at the periphery (upper panel; scale bar, 150 μm) and center of implant (lower panel; scale bar, 80 μm). (TIF) [file pone.0125697.s004.tif]

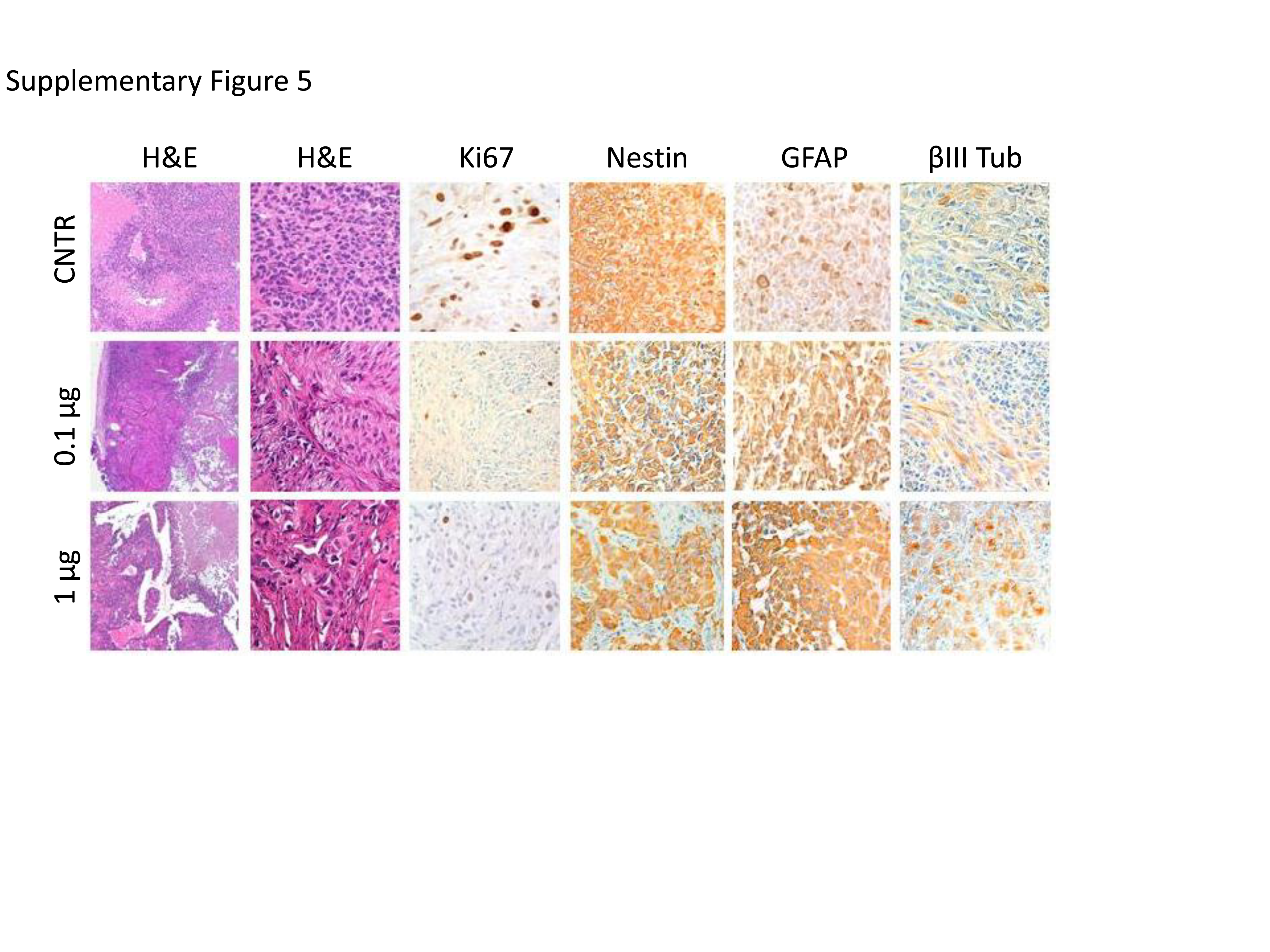

Supplement: S5 Fig — Three weeks after the last treatment, control tumors (CNTR) or tumors treated with BMP7v (1 μg or 0.1 μg) were assessed for morphology by H&E staining. In addition, tumor cell proliferation was analysed by Ki67, and tumor cell differentiation was assessed by nestin, GFAP, and βIII tubulin (βIII Tub) immunohistochemistry. (TIF) [file pone.0125697.s005.tif]

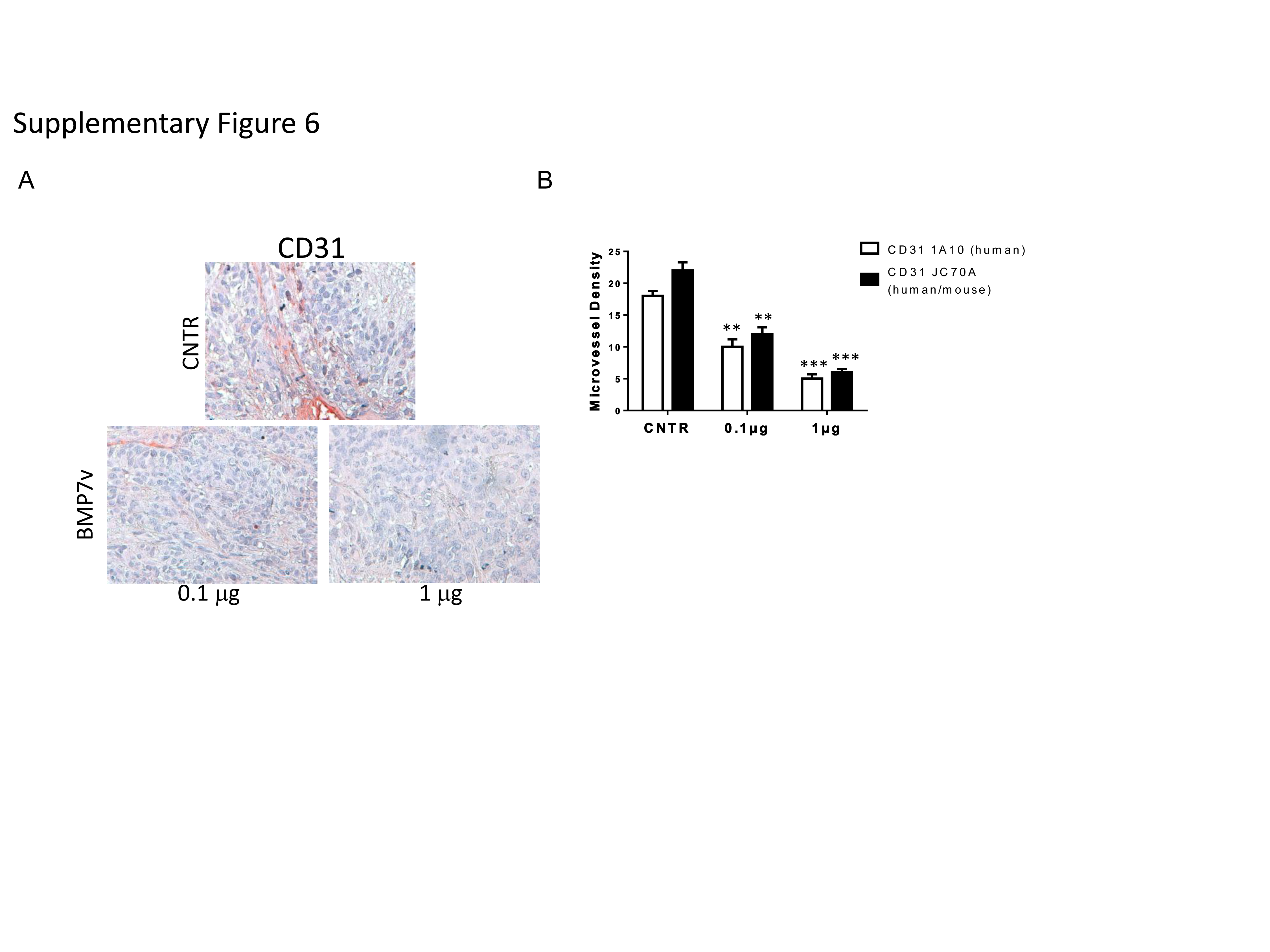

Supplement: S6 Fig — (A) Immunohistochemistry was performed on vehicle (CNTR) or BMP7v treated (0.1 μg or 1 μg) tumors three weeks following treatment using a human-specific CD31 antibody. (B) Graph represents MVD assessed from both human and mouse CD31 immunostaining, and asterisks denote statistically significant (**, p<0.05; ***, p<0.001) differences compared to vehicle controls (CNTR). (TIF) [file pone.0125697.s006.tif]
