# Supplementary material for: A BMP7 Variant Inhibits Tumor Angiogenesis In Vitro and In Vivo through Direct Modulation of Endothelial Cell Biology
Source: PLoS One. 2015 Apr 28;10(4):e0125697. doi: 10.1371/journal.pone.0125697 (PMC4412825; doi:10.1371/journal.pone.0125697)
Supplement: S1 Table — Quantitative PCR analysis was performed, and data was normalized to the geometric mean of at least three housekeeping genes. ND, not determined. Changes in red denote p<0.05. (DOCX) [file pone.0125697.s007.docx]

Supplementary Table 1

|  | |  |  |  |  |
| --- | --- | --- | --- | --- | --- |
|  | **Taqman™** | | | **ELISA** | |
|  | **HUVEC** | | **ECFC** | **HUVEC** | **ECFC** |
| **Gene** |  | |  |  |  |
| cKit | **-28.4** | | **-35.3** | ND | ND |
| FGFR1 | **-1.27** | | **-1.24** | ND | ND |
| VEGFR2 | **-2.5** | | **-2.7** | **-1.6** | **-3.5** |
| Kit ligand | **-2.9** | | **-3.5** | ND | ND |
| MCP-1 | **-21.1** | | **-25** | **-8.4** | **-8.2** |
| MMP1 | **-3.2** | | **-3.2** | ND | ND |
| PECAM-1 | **-2.9** | | **-2.4** | ND | ND |
| PlGF | **-32** | | **-7.4** | **-11.6** | **-5.4** |
