# Supplementary material for: A BMP7 Variant Inhibits Tumor Angiogenesis In Vitro and In Vivo through Direct Modulation of Endothelial Cell Biology
Source: PLoS One. 2015 Apr 28;10(4):e0125697. doi: 10.1371/journal.pone.0125697 (PMC4412825; doi:10.1371/journal.pone.0125697)
Supplement: S2 Table — (DOCX) [file pone.0125697.s008.docx]

Supplementary Table 2

| **GSLC1** | **Control**  **(*n*, 6)** | **BMP7v**  **(*n*, 6)** | ***p*** |
| --- | --- | --- | --- |
| **Periphery**   - no. vessels - vascular area (μm^2^) - vascular area (% total) | 17 + 2.2  108452 + 11602  7.71 + 1.21 | 18 + 2.1  35743 + 3593  2.17 + 0.22 | **ns**  **0.021**  **0.019** |
| **Center**   - no. vessels - vascular area (μm^2^) - vascular area (% total) | 18.5 + 2.49  28187 + 4012  1.71 + 0.24 | 11.75 + 1.46  13588 + 3144  0.79 + 0.19 | **0.042**  **0.017**  **0.015** |
| **GSLC28** | **Control**  **(*n*, 5)** | **BMP7v**  **(*n*, 5)** | ***p*** |
| **Periphery**   - no. vessels - vascular area (μm^2^) - vascular area (% total) | 32 + 3.51  108398 + 3331  6.62 + 0.22 | 28.1 + 1.30  79519 + 10083  4.91 + 0.61 | **ns**  **0.026**  **0.031** |
| **Center**   - no. vessels - vascular area (μm^2^) - vascular area (% total) | 35.8 + 4.4  63397 + 5755  4.42 + 0.46 | 23.2 + 2.0  40049 + 6087  2.48 + 0.37 | **0.031**  **0.024**  **0.012** |
